# HPTLC Combined with sHetCA and Multivariate Statistics for the Detection of Bioactive Compounds in Complex Mixtures

**DOI:** 10.3390/molecules29246027

**Published:** 2024-12-20

**Authors:** Vaios Amountzias, Evagelos Gikas, Nektarios Aligiannis

**Affiliations:** 1 Department of Pharmacognosy and Natural Products Chemistry, Faculty of Pharmacy, National and Kapodistrian University of Athens, 15771 Athens, Greece; aligiannis@pharm.uoa.gr; 2 Department of Analytical Chemistry, Faculty of Chemistry, National and Kapodistrian University of Athens, 15771 Athens, Greece

**Keywords:** HPTLC, chemometrics, DPPH scavenging activity, HetCA, multivariate analysis, analytical chemistry, natural products, FCPC, artificial extract

## Abstract

High-Performance Thin Layer Chromatography (HPTLC) is widely utilized in natural products research due to its simplicity, low cost, and short total analysis time, including data treatment. While bioautography can be used for rapid detection of bioactive compounds in extracts, the number of available bioautographic methods is limited mainly due to the high cost and difficulty in developing protocols that lead to accurate and reproducible results. For this reason, an alternative method for the detection of bioactive compounds in plant extracts prior to their isolation using HPTLC, combined with multivariate chemometrics, was previously explored by our lab. To evaluate this method and compare it to other chemometrics-based methods, an artificial mixture (ArtExtr) of 59 standard compounds was used as a case study. The ArtExtr was fractionated by FCPC and the inhibitory activity of all fractions against DPPH was evaluated, while their chemical profiles were recorded using HPTLC. Multivariate statistics and the heterocovariance approach (HetCA) were employed and compared, with the success rate in detecting the ArtExtr bioactive substances being 85.7% via sparse heterocovariance (sHetCA). HPTLC combined with sHetCA can serve as a valuable tool for the detection of bioactive compounds in complex mixtures when bioautography is not feasible.

## 1. Introduction

High-Performance Thin Layer Chromatography (HPTLC) is a widely used, simple, and rapid chromatographic technique in natural products research. Although commercial systems can be costly, the low volume of solvents required, as well as the significantly shorter analysis time compared to other chromatographic methods, render HPTLC an excellent tool for the study of natural products. The use of specialized equipment ensures precise sample application and detection instrumentation, such as densitometers, allows for accurate evaluation of the acquired densitograms. Additionally, the captured chromatogram images can be stored and analyzed retrospectively. The high cost of commercial HPTLC systems has prompted the development of open-source equipment and alternative software [1,2]. In recent years, various open-source software, such as rTLC [3] and DE-TLC [4], as well as smartphone applications [5], have been created to make the detection step in HPTLC more affordable [6,7,8]. These tools can convert digital photographs of HPTLC plates into densitograms, effectively simulating the operation of a scanner.

Numerous qualitative and quantitative analysis methods have been developed for HPTLC, while its hyphenation with other techniques, such as mass spectrometry (MS), offers great potential for the detection of individual compounds in complex mixtures [9,10,11]. Key advantages of HPTLC include the high sample throughput, as multiple samples can be analyzed in a single run, the simple two-dimensional metabolite separation, the flexible detection strategies based on UV–Vis, FLR detection or derivatizing reagents, and the ability to simply combine with biological detection methods, known as HPTLC-bioautography [12,13]. The development of databases containing spectra (UV–Vis and FLR) of standard compounds and their color properties at various wavelengths prior to and after derivatization, as well as the application of annotation methods, have greatly aided the identification of secondary metabolites in various extracts [11,14].

HPTLC-bioautography combines the separation of multiple samples concurrently with effect-directed enzymatic or biological assays, offering a significant advantage for the rapid detection of bioactive compounds. Several protocols have been developed for the bioautographic screening of active metabolites, such as antimicrobial, antioxidant, and against enzyme-inhibition assays (e.g., tyrosinase) [15,16,17,18,19]. However, the number of available bioautographic methods is rather limited compared to the in vitro methods. Some biological assays, such as cytotoxicity ones, require cell lines that cannot be easily employed in HPTLC-bioautography. Furthermore, the cost of certain existing bioautographic protocols can be higher than the corresponding in vitro methods, since they often necessitate large quantities of reagents (e.g., enzymes, substrates etc.) for the detection of metabolites across an entire HPTLC plate.

The need for rapid detection of secondary metabolites in extracts prior to isolation has led to the development of powerful analytical techniques that enable high-throughput screening and dereplication approaches [20,21,22,23]. Liquid chromatography hyphenated with MS [24,25,26], as well as nuclear magnetic resonance (NMR), have predominantly been used as a technique, combined with chemometric [27,28] or heterocovariance (HetCA) methodologies [29,30,31,32,33,34], while UPLC-DAD has also been utilized for this purpose [35].

Multivariate chemometrics have been successfully applied by our group in the past for the detection of secondary metabolites present in the methanolic extract of *Morus alba*, that were active against tyrosinase, using HPTLC [36]. Two methodologies were developed and tested, one based on peak picking and the other on densitogram binning. After implementing the chemometric approaches, the compounds oxyresveratrol, *trans*-dihydromorin, morucin, and kuwanon C were identified in the fractions by comparison with the isolated compounds. While both methodologies yielded comparable results, a thorough evaluation is needed to determine the success rate of each approach and optimize the methods.

Following the aforementioned promising results, the meticulous evaluation of the multivariate statistical analysis, as well as the development of new approaches (sHetCA), needed to be accomplished in a controlled environment. In our previous work, an artificial complex mixture of 59 standard compounds (artificial extract, ArtExtr) was prepared as a case study to simulate a plant extract [37]. The ArtExtr was fractionated using Fast Centrifugal Partition Chromatography (FCPC), and the fractions’ DPPH radical scavenging activity was evaluated. This bioassay was selected due to its reproducibility, simplicity, ability to quantify the results, availability of existing data in the literature, and low cost. Additionally, the cumulative effect of the substances is primarily observed in this assay [38]. In the current study, these data were utilized for the implementation of chemometrics-based methodologies, using the HPTLC profiles of the fractions. First, a comparison between different software for converting HPTLC chromatogram photographs into densitograms was conducted. Both manual peak picking and densitogram binning approaches were evaluated after the implementation of HetCA and multivariate statistics methodologies, while a comparative study between the different chemometric methods was performed to determine the success rate of each method. In this study, we conducted a systematic and thorough investigation of the application of chemometric analyses to a complex artificial mixture of known compounds with diverse chemical properties under controlled conditions. This work aimed to explore potential challenges that may arise during the analysis of plant extracts and propose alternative approaches to HPTLC-bioautography for the detection of bioactive compounds prior to their isolation, mainly when bioautography is not feasible. It should be noted that the Supplementary Materials contains information beyond listing purposes and should be seen as an integral part of the main text.

## 2. Results and Discussion

### 2.1. Artificial Extract (ArtExtr) Preparation and Fractionation

The ArtExtr (Table 1) was prepared using 59 standard compounds, to approximate the composition of a plant extract (Table S1, Figure S1). The mixture was fractionated using FCPC and the resulting fractions were evaluated for their DPPH radical scavenging activity in vitro (Figure S2). The chemical profiles of the fractions were assessed through normal and reverse phase (NP and RP, respectively, Figure S3) HPTLC, as well as ^1^H-NMR (Figure S4). The ArtExtr preparation and FCPC fractionation, as well as the evaluation of the fractions’ DPPH scavenging activity and chemical profiles through ^1^H-NMR were all previously reported by Amountzias et al. [37].

### 2.2. Preliminary Chromatographic Data Evaluation

An analysis was conducted to identify the substances in the chromatograms of the ArtExtr fractions based on the chromatographic (HPTLC) and spectral (^1^H-NMR) data of the standard compounds (Figures S5–S7). However, it is noteworthy that ten substances were not detected in HPTLC analysis. Among them, palmitic acid (**08**) lacks chromophore groups [39] and does not react with the sulfuric vanillin reagent, rendering its detection impossible under the employed detection conditions. The inability to annotate compounds **10** (caffeic acid), **19** (thymol), **29** (homovanillic acid), **39** (3,5-dihydroxybenzoic acid), and **49** (*p*-hydroxyphenylacetic acid) was due to the fact that they exhibited the same or similar Rf value as other substances in the same fractions, which complicated their separation. The remaining compounds **01** (galanthamine hydrobromide), **20** (oxytetracycline hydrochloride), **31** (tannic acid), and **40** (D-(−)-quinic acid) were not detected using NMR either, and are characterized by limited solubility in organic solvents. Therefore, it was concluded that the formation of a precipitate occurred during the introduction of the initial mixture into the FCPC separation column, which contained water as the stationary phase (Table S2). This, coupled with the high water solubility of certain compounds, led to the elution of some substances in the extrusion fraction at the end of the FCPC separation process. Furthermore, compound **31** is a mixture of polygalloyl glucoses or polygalloyl quinic acid esters, making its detection even more challenging [37]. For these reasons, they were not included in the study. Regarding compounds **09** (reserpine), **12** (ephedrine), and **13** (harmine), they were not detected using NMR, but they were identified by UV–Vis detection. This spectroscopy exhibits significantly higher sensitivity compared to NMR. All three substances contain nitrogen and extensively *π*-conjugated systems, resulting in a very high molar absorption coefficient. Consequently, they are able to absorb in the UV–Vis spectrum even at very low concentrations.

### 2.3. Selection of Software for Converting HPTLC Chromatogram Photographs into Densitograms

The HPTLC plates were photographed at 254 nm, 366 nm, and under white light after derivatization with the sulfuric vanillin reagent, employing the CAMAG Visualizer 2 (CAMAG, Muttenz, Switzerland). VisionCats 3.0 (CAMAG, Muttenz, Switzerland) [11] and rTLC v.1.0 [3] were evaluated for the conversion of the digital images into densitograms, based on the following principle.

Digital images are composed of individual picture elements known as pixels, arranged in rows and columns. As a result, a digital image can be considered as a numerical matrix. The software applications mentioned traverse this matrix, using a moving average algorithm to create a graphical representation. They calculate the average horizontally for each pixel of the chromatogram across the RGB channels (R: red, G: green and B: blue), as well as the grayscale, which is the average of these three channels. To generate the densitogram, the background is defined as the maximum value, while a decrease in its intensity corresponding to a dark spot at 254 nm, or a colored spot under white light. Conversely, at 366 nm, the background is black and, as such, it is considered as the minimum value, so an increase in its intensity corresponds to a colored spot [3,11].

**Figure 1:**
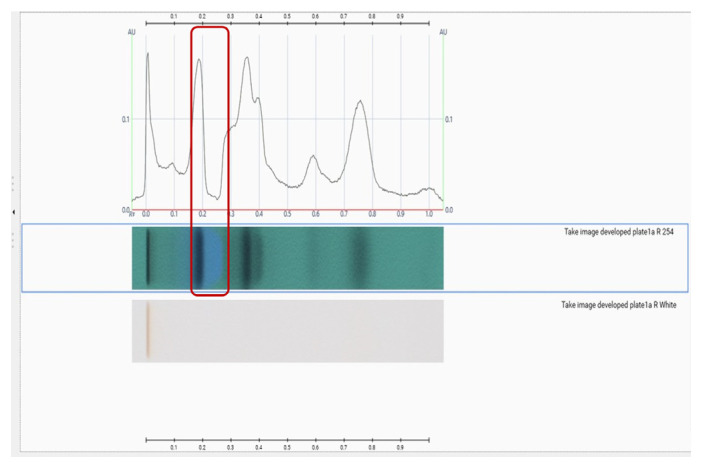
Example of a densitogram of fraction Fr54, in RP at 254 nm, via VisionCats 3.0 software. In the red frame, the lack of peak of the blue spot at Rf = 0.2–0.3 is highlighted.

VisionCats 3.0 was initially used to convert the chromatogram photographs into densitograms, which utilizes the grayscale to generate the plot [11]. However, an issue was encountered during this process. For some substances, such as the spot of blue color depicted in Figure 1, no peaks were observed in the densitogram, while the baseline appeared visibly distorted. The reason for the absence of a peak for this substance is that it has the same color as the background (blue) at 254 nm. In this case, there is no decrease in absorption in order for it to be identified as a peak, but rather the absorption is maintained in the same level or increased. This results in the substance appearing as a negative peak. Since this software does not recognize negative peaks, it adjusts the baseline to keep those peaks on the positive Y-axis, causing unexpected distortions in the densitogram.

To address this particular issue, the free rTLC v.1.0 [3] software was utilized. This software allows for the selection of specific RGB channels, as deemed necessary for each case. Employing this approach, the green channel was selected to generate densitograms at 254 nm in NP, while the blue channel was chosen for 254 nm in RP, as well as 366 nm of both phases. Additionally, the grayscale was selected to generate densitograms before and after derivatization with the sulfuric vanillin reagent. The specific channel selections were made for the following reasons:

- For NP HPTLC plates, the green channel was selected to generate densitograms at 254 nm, as the plates appear green at this wavelength and the green channel best captures the peak intensity;
- The blue channel was selected to generate the densitograms of the RP plates at 254 nm, due to their color, and to avoid the issues encountered with the VisionCats 3.0 software, as aforementioned (Figure S8);
- The blue channel was also selected to generate densitograms at 366 nm, as most substances appeared blue in color at this wavelength;
- Finally, the grayscale was selected for generating densitograms prior to and after derivatization with the sulfuric vanillin reagent, as the various colors resulting from the reactivity of the substances with the reagent are best captured using the grayscale.

The densitogram images were processed by extracting points with a pixel width of 1000, corresponding to the total height of the plate. After removing the points that did not belong to the densitogram, 687 Rf points were obtained per sample. This resulted in Excel CSV files for each wavelength and phase, with the data in a two-dimensional matrix format featuring samples as rows and Rf values as columns. The data were then post-processed using the free Fityk v. 1.3.1 software [40] to perform baseline correction using the cubic spline method. Subsequently, each chromatogram was processed in Excel to invert the densitograms, ensuring the peaks were positioned on the positive Y-axis. This was achieved by multiplying negative values by −1. In cases where negative peaks were present, such as in fraction Fr54 (Figure S8), the baseline correction facilitated their conversion into positive peaks (Figure S9).

### 2.4. Comparative Study of Chemometric Methods for Identifying DPPH-Scavenging Compounds Using HPTLC

After the aforementioned procedure, two approaches were used to analyze the densitograms: the peak integration method and the “blind” method. In the first approach, the integration values (AUC, areas under the curve) of the densitograms peaks were evaluated, while in the second, the binning method was applied. Initially, a comparative study of the two methodologies was conducted to identify substances with DPPH scavenging activity, highlighting the advantages and disadvantages of each approach using a simple bioassay, where the cumulative activity of the components is mainly observed.

#### 2.4.1. Peak Integration Method of HPTLC Densitograms for the Detection of Compounds with DPPH Scavenging Activity

**Figure 2:**
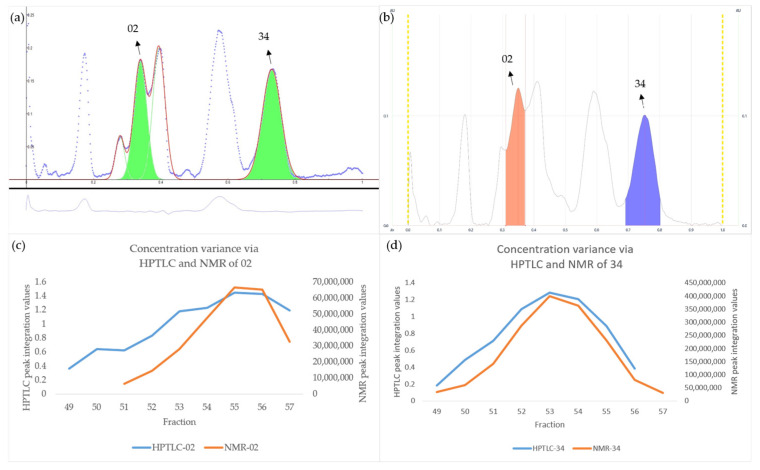
(**a**) Example of peak integration of compounds **02** and **34** (quercitrin and gallic acid, respectively) in the densitogram of fraction Fr52, in RP, at 254 nm using Fityk v. 1.3.1. The blue dashed line represents the densitogram, the green lines indicate the integration regions, and the red lines show the overall integration model; (**b**) example of peak integration of the same peaks using the VisionCats 3.0 software. The blue line represents the densitogram and the yellow dashed lines represent the start and end of the densitogram; comparison of HPTLC and NMR integration values of (**c**) **02** and (**d**) **34** in fractions Fr49-57.

As aforementioned, the identification of the compounds in the HPTLC chromatograms was based on the comparison with the chromatographic data of the standard substances (i.e., Rf values and colors at 254 nm, 366 nm, under white light after derivatization with the sulfuric vanillin reagent and at 366 nm post-derivatization), as well as the comparison of their spectral data. While this matching process facilitated rapid evaluation of the results, it did not bias the prediction of their contribution, as random code names could have been assigned to the identified substances. Peak integration of the densitograms was performed at one wavelength for each substance using the Fityk v. 1.3.1 software. For the integration of the peaks of the identified substances, all wavelengths of both phases prior to and after derivatization were considered, assuming the complete separation of these compounds, as well as the presence of sufficient intensity to produce a detectable peak in the densitogram (Figure 2a, **34**; gallic acid). In cases where the peaks were not completely separated, peak deconvolution was performed using Fityk. In this deconvolution process, the blue dashed line represents the densitogram, the green lines indicate the integration regions, and the red lines show the manual curve fitting (Figure 2a, **02**; quercitrin). In Figure 2b, the integration method of the same peaks using VisionCats 3.0 is presented, where no deconvolution is performed. It should be emphasized that the integration of the peaks did not occur at the maximum wavelength (*λ*_max_) for each compound; therefore, the actual concentrations may differ in absolute value. However, they maintain a corresponding variance, as shown in Figure 2c,d, where the HPTLC and NMR integration values of compounds **02** and **34** (quercitrin and gallic acid, respectively) appear to be highly correlated, with correlation coefficients of 0.94 and 0.98, respectively.

Of the 49 identified substances, 43 were integrated. The peaks of the remaining six substances, namely, compounds **04**, **25**, **30**, **44**, **51**, and **56** (kaempferol, sucrose, ellagic acid, isoferulic acid, aucuboside, and baicalein, respectively) could not be integrated. The reason was the overlapping with other substances (**04**, **44** and **56**) or the unsuitability of the specific development system for these compounds (Rf**_25_** _and **51**_ = 1.0, Rf**_30_** = 0.0) (Table S3).

Based on the above, 43 substances were included in this study, 14 of which exhibited active DPPH scavenging activity in the in vitro assay (Table S1). Subsequently, a table was created with the integration values of the densitogram peaks, which was used to perform statistical analysis (Integr. matrix). Due to the presence of several missing values, since each compound was only present in specific fractions and absent from the rest, these values were replaced with 0 (Integr. matrix-0). Two methodologies were employed for the statistical processing of the results: the HetCA study of the chromatographic data and bioactivity, and the multivariate analysis.

##### HetCA Study of the Chromatographic Data with the DPPH Scavenging Activity

The HetCA was performed using the Integr. matrix-0 through the free Data Fusion-based Discovery (DAFdiscovery) platform [41]. This platform’s key feature is the STOCSY (statistical total correlation spectroscopy) algorithm, which calculates the covariance and correlation between a specific variable across all samples and all other variables present [42]. Option 4 of DAFdiscovery was used, where the MS data are correlated with the bioactivity. In this study, the data from the integration of the HPTLC peaks were processed in an Excel CSV file as MS data and correlated with the DPPH scavenging activity.

The results are presented in Figure S10, where the substances with the highest correlation with the activity are shown in red, while those with the lowest correlation are shown in blue. The circle size indicates the covariance with the activity. Compounds that had a positive correlation with the activity were considered as active. The comprehensive results are presented in Table 2 and Table S4.

The second method used was based on the correlation and covariance algorithms built into Excel (Microsoft Office 2016 Pro Plus VL x64, Microsoft Corporation, Redmond, WA, USA). The values were calculated for each compound only in the fractions where it was detected, using the unmodified integration matrix (Integr. matrix). This particular approach can be characterized as “sparse heterocovariance approach (sHetCA)” of chromatographic data and bioactivity, since it is focused on the “important” data, i.e., the integration values of the substances, rather than including missing data, to determine the covariance and correlation of each compound with the bioactivity. Substances that presented positive correlation coefficients and covariance at the same time were considered active. The efficiency of this method was improved when substances in the first fractions (Fr02-19) were not included (sHetCA DPPH Fr20-70), since these fractions exhibited negligible DPPH scavenging activity (<5%). This exclusion prevented the false positive prediction of the compounds **18** and **52** (shikonin and sclareol, respectively) in those low-activity fractions, which had presented positive correlation and covariance, but made no significant contribution to the overall activity. In total, six substances were not included in this procedure, two of which were initially predicted as active incorrectly. The comprehensive results are presented in Table 2 and Table S4.

##### Multivariate Analysis of Chromatographic Data for the Detection of Compounds with DPPH Scavenging Activity

In parallel with the HetCA method, multivariate analysis of the chromatographic data was performed to identify compounds with DPPH scavenging activity, using the Integr. matrix-0. The initial stage for statistical processing involved centering and scaling the data. In this study, the Pareto and Unit Variance (UV) scaling methods were examined. No scaling was not used since the peaks integration was not performed at *λ*_max_ for each substance. Therefore, the integration values are not comparable quantitatively in order to investigate the possibility that the obtained results are due to the concentration of each ingredient. In addition, a comparison of the results between PLS and OPLS models was carried out. All resulting models exhibited satisfactory fitting capacity (R^2^) and predictive (Q^2^) ability (Table 3).

In order to validate the models and ensure that they were not randomly generated, permutation analysis was performed with 200 permutations for each model to verify their good predictive and fitting ability. All resulting models showed much lower Q^2^ and R^2^ values compared to the original models, confirming that the original models can effectively predict the required scenarios and do not simply produce random results (Figure S11). Furthermore, various diagnostic measurements were performed to ensure the validity of the models. Scatterplots of predicted versus observed values for the resulting models showed linear regression. The root mean square error of estimate (RMSEE) for DPPH PLS Pareto scaling (DPPH PLS Par), DPPH OPLS Pareto scaling (DPPH OPLS Par), DPPH PLS UV scaling (DPPH PLS UV), and DPPH OPLS UV scaling (DPPH OPLS UV) models were 12.32, 6.89, 11.40, and 6.93, respectively, indicating that the constructed models were accurate and able to estimate and predict the bioactivity of the substances in the fractions (Figure S12). In addition, the implementation of Hotelling T^2^Range (Figure S13) and DModX (Figure S14) did not show significant outliers in the fractions. The normal probability plot of residuals showed a linear trendline, suggesting that the residuals are randomly distributed (Figure S15). The analysis and comparison between the scatterplots of the scores and the cross-validation scores (CV-Scores) demonstrated the stability of the observations (fractions) (Figure S16). Small changes between the two plots indicate a model that is stable to the inclusion or exclusion of observations. Finally, the cross-validation analysis (CV-ANOVA) [43] showed that the DPPH PLS Par, DPPH OPLS Par, DPPH PLS UV, and DPPH OPLS UV models had *p*-values of 2.16 × 10^−22^, 3.16 × 10^−24^, 2.64 × 10^−23^, and 2.03 × 10^−25^, respectively, much lower than 0.05. Overall, the diagnostics of the models demonstrated their validity.

The evaluation of the active substances against DPPH was performed by examining the coefficient plots (Figure S17) and the VIP plots (Figure S18). The variables considered to contribute to the activity were those with positive MLR (CoeffMLR) and correlation (p(corr)) coefficients, simultaneously, for the coefficient plots. For the VIP plots, the active variables were those with VIP coefficients above 0.6 and positive correlation coefficients, simultaneously. The results are presented in Table 2.

It is worth noting that the application of multivariate analysis to fractions Fr19-70 led to more false negative results. The reason was the decrease of the activity variance caused by the exclusion of the fractions with low activity. Additionally, reducing the data used to perform multivariate analysis resulted in increased standard deviations, decreased statistical power, and reduced precision [44].

##### Comparison Between the HetCA and Multivariate Statistics Methods of the Peak Integration Method for the Detection of Compounds with DPPH Scavenging Activity

The main difference between the sHetCA method using Excel and the HetCA method using the DAFdiscovery platform, as well as the multivariate analysis, is that missing values are not taken into account when calculating correlation coefficients and covariance in sHetCA. As aforementioned, the presence of the substances in specific fractions leads to several missing values in the integration table for each substance. Replacing these missing values with zeros, in order to perform the HetCA study using the DAFdiscovery platform, as well as the multivariate analysis, can alter the calculated values, as shown in the example of Table 4.

The active substance **23** (curcumin) was detected in fractions Fr20-24. This results in having zero values in 64 fractions in the Integr. matrix-0, while the true integration values are found in only 5 fractions. This disparity in the number of fractions with non-zero values causes a change in the mean integration value. According to the equations for calculating the covariance (1) and correlation coefficient (2), performing the calculations on all the fractions would lead to the prediction of compound **23** as inactive, as observed in the DAFdiscovery platform results (Table S4). However, this prediction changes when the calculations are performed using only the fractions in which the specific substance is actually detected (Table 4 and Table S4).

(1) $$COV_{x,y}=\frac{\sum \left(x_{i}-\bar{x}\right)\left(y_{i}-\bar{y}\right)}{N-1}$$

where *cov_x,y_* is the covariance between variables *x* and *y*, $\bar{x}$ is the mean of *x* values, $\bar{y}$ is the mean of *y* values, and *N* is the number of data values.

(2) $$\rho_{x,y}=\frac{\mathrm{cov}\left(x,y\right)}{\sigma_{x}\sigma_{y}}$$

where *ρ_x,y_* is the correlation between variables *x* and *y*, *cov_x,y_* is the covariance between variables *x* and *y*, and *σ_x_* and *σ_y_* are the standard deviations of *x* and *y*, respectively.

Although the Integr. matrix-0 was used to calculate the correlation coefficients and/or the covariance through both the DAFdiscovery platform and the DPPH OPLS Par CoeffMLR model, the latter being the model with the best results from the multivariate analysis study, these two methods employ different mathematical formulas to derive the results. The first method directly calculates these values from the integration values in the set of fractions, while the second is based on the method of projections and the extrapolation of principal components aimed at achieving the best possible correlation between variables X (peak integration values) and Y (DPPH scavenging activity) [45,46]. Thus, despite the fact that the results obtained by these two methods are comparable, there are several differences between them (Table S4). Due to the way the covariance and the correlation coefficients are calculated via the DAFdiscovery platform, which has been shown to lead to some erroneous results, this method was rejected.

Comparing the results of DPPH OPLS Par CoeffMLR with those of sHetCA through Excel, the two methods showed comparable performance, with OPLS Pareto being slightly better in terms of false negative results (1 vs. 2), while the sHetCA was slightly better in terms of false positive results (10 vs. 11). Both methods were found to be effective and suitable for the detection of bioactive substances using HPTLC. Nevertheless, different datasets require the development of different models and the assessment of different plots to predict bioactive compounds. For instance, the DPPH OPLS Par Coef and DPPH OPLS UV VIP models both correctly predicted the same number of bioactive compounds (13), but differed significantly in the number of false positive results (11 vs. 15). While conducting diagnostic analyses on the models provided some insight for the exclusion of some of them, more extensive studies, such as Monte Carlo cross-validation, are needed to quickly assess the predictive ability of the models [47]. This diagnostic process is an additional step that requires time. In contrast, the sHetCA study produced reliable results, with fewer false positives and the analysis time was much shorter, since a correlation matrix was initially created using the Integr. matrix and covariance was calculated only for substances with a positive correlation coefficient with the activity. Therefore, although multivariate statistical analysis can be used to identify bioactive substances by HPTLC, the sHetCA method was preferred.

##### False Negative Results from the Peak Integration Method for the Detection of Compounds with DPPH Scavenging Activity

The active substances that were incorrectly predicted by the sHetCA method were compounds **02** and **46** (quercitrin and sinapic acid, respectively), while **06** (resveratrol) was predicted incorrectly by the DPPH OPLS Par CoeffMLR model.

**Figure 3:**
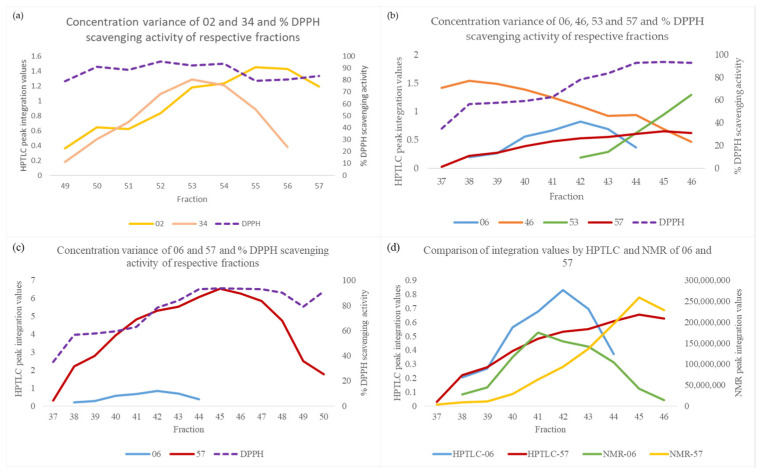
(**a**) Concentration variance of compounds (**a**) **02** and **34** (Fr49-57); (**b**) **06**, **46**, **53,** and **57** (Fr37-46); (**c**) **06** and **57** (Fr37-50) and % DPPH scavenging activity of respective fractions; and (**d**) comparison of integration values by HPTLC and NMR of compounds **06** and **57** in fractions Fr37-46.

Regarding compound **02**, it was detected in fractions Fr49-57, while the covariance and the correlation coefficient with the DPPH scavenging activity were −1.07 and −0.60, respectively. From the plot in Figure 3a, it is observed that the variance in the concentration of **02** is similar to that of the activity in fractions Fr49-54. In fact, the correlation coefficient and the covariance for these fractions are 0.74 and 1.24, respectively. However, their values change due to the large difference observed in fractions Fr55-57. The activity of fractions Fr49-54 ranges from 78.9% to 93.5%, while it decreases in fraction Fr55 (79.4%). This decrease is attributable to the lower concentration of the active compound **34** (gallic acid), which is the major compound in the respective fractions and overshadows the activity of **02**.

Compound **46** was detected in fractions Fr37-46, while the covariance and the correlation coefficient with the DPPH scavenging activity were −5.70 and −0.88, respectively. In the plot of Figure 3b, it is observed that **46** contributes to the activity of fractions Fr37-40, with a correlation coefficient and covariance of 0.30 and 0.18, respectively, for these fractions. However, in fractions Fr41-46, the concentration of this substance decreases, while the concentrations of the active substances **06** and **57** (resveratrol and 6,7-dihydroxycoumarin, respectively) increase. The integration values of **57** were scaled down for better visualization in the plot of Figure 3b. Additionally, the active substance **53** (protocatechic acid) begins to elute in fraction Fr42. Compounds **06**, **53,** and **57** are the major metabolites in the respective fractions, and are responsible for the increasing activity in these fractions. The cumulative activity of these compounds overlaps that of **46** in fractions Fr41-46, resulting in its incorrect prediction.

Finally, compound **06** had a negative CoeffMLR coefficient with the activity, while it had a positive correlation coefficient. The calculations were performed using the Integr. matrix-0, so the integration values from the set of the fractions was used. Compound **06** was detected in fractions Fr38-44. In the same fractions, the active substance **57** (Fr37-50) was also detected. Figure 3c depicts the HPTLC integration variance of these substances in relation to the activity, while in Figure 3d, a comparison is made between the integration variance via HPTLC and NMR, where the HPTLC integration values of compound **57** were normalized to be on the same scale as those of **06** for better visualization. Many substances containing chromophore groups in their molecule emit photons in a wide spectrum after being excited at 366 nm. One such example is coumarins, such as **57**, which bear extensive *π*-conjugated systems and have a high molar absorption coefficient [48]. The peaks of compound **57** were integrated at 366 nm. As evident from Figure 3c,d, the integration at this wavelength exaggerated the true concentration of the substance, especially in fractions Fr38-43, in which **06** was detected, overshadowing its activity.

##### False Positive Results from the Peak Integration Method for the Detection of Substances with DPPH Scavenging Activity

The compounds whose activity was overestimated by both the DPPH OPLS Par CoeffMLR and sHetCA were **05** (phlorizin), **09** (reserpine), **12** (ephedrine), **13** (harmine), **32** (caffeine), **55** (loganin), and **58** (umbelliferone). Meanwhile, compounds **07** (aristolochic acid), **15** (naringenin), and **47** (vanillic acid) were overestimated only in the sHetCA method, while compounds **17** (nicotinic acid), **28** (*p*-coumaric acid), **43** (*m*-coumaric acid), and **59** (colchicine) were overestimated solely in the DPPH OPLS Par CoeffMLR model.

**Figure 4:**
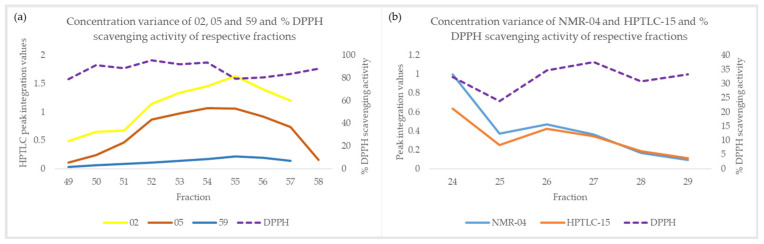
(**a**) Concentration variance of compounds (**a**) **02**, **05,** and **59** (Fr49-58), and (**b**) NMR-**04** and HPTLC-**15** (Fr24-29) and % DPPH scavenging activity of respective fractions.

In most cases, substances with overestimated activity showed high concentration covariance with some of the active compounds. The same conclusion was also drawn in the NMR-HetCA study [37]. For example, the concentrations of compounds **05** and **59** were similar to that of the active compound **02** (quercitrin) (correlation was 0.97 and 0.99, respectively), and the concentration of the non-active compound **15** was comparable to that of active compound **04** (kaempferol) (Figure 4). Similarly, most of the other substances whose activity was overestimated showed high covariance and correlation coefficients with active compounds present in the respective fractions (Figure S19). The correlation coefficient values are shown in Table 5, where the left vertical column lists the compounds that were falsely predicted as active, and the upper horizontal row shows the active compounds with which they exhibit high correlation coefficients. Active compounds **04** and **56** could not be integrated in the HPTLC densitograms due to overlapping, so the integration values from ^1^H-NMR were used for the calculations. Regarding compounds **09**, **12,** and **13**, they were not detected in the ^1^H-NMR spectra, but were detected in HPTLC due to their high molar absorption coefficient, despite their actual concentrations being very low.

Compounds **07**, **13**, **17,** and **58** did not exhibit any covariance in their concentration with active compounds. The reason for their overestimated activity is the incidental correlation and covariance with the overall activity, which was influenced by the presence of several active compounds, as mentioned below.

**Figure 5:**
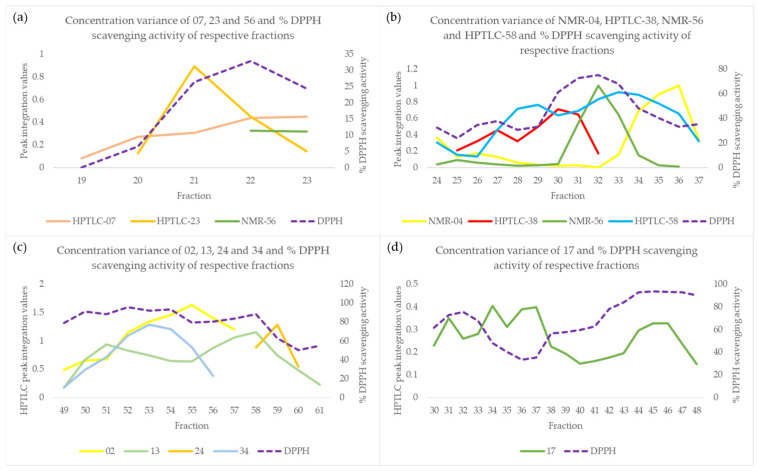
Concentration variance of compounds (**a**) **07**, **23,** and **56** (Fr19-23); (**b**) NMR-**04**, HPTLC-**38**, NMR-**56,** and HPTLC-**58** (Fr24-37); (**c**) **02**, **13**, **24,** and **34** (Fr49-61); and (**d**) **17** (Fr30-48) and % DPPH scavenging activity of respective fractions.

Concerning compound **07**, it was detected in fractions Fr19-23. In fractions Fr20-23 and Fr22-23, respectively, the active substances **23** and **56** (curcumin and baicalein, respectively) are present. As shown in the plot of Figure 5a, the variance in the concentration of compound **07** in these fractions is similar to the variance of the overall activity, which can be attributed to the presence of compounds **23** (Fr20-23) and **56** (Fr22-23). Compound **58** (umbelliferone) was detected in fractions Fr24-37, where the active substances **04** (kaempferol, Fr24-37), **38** (catechol, Fr25-32), and **56** (baicalein, Fr24-36) were also present. As depicted in the plot of Figure 5b, the concentration variance of compound **58** in these fractions appeared similar to the variance of the overall activity. However, this similarity in variance was actually due to the presence of the active compounds **04**, **38**, and **56**, rather than compound **58** itself. The covariance of compound **13** (Fr49-61) with the activity (Figure 5c) appears to be due to the presence of the active compounds **02** (Fr49-57), **24** (Fr58-60), and **34** (Fr49-56). Finally, **17** was detected in fractions Fr30-48 (Figure 5d). The reason for the overestimation of its activity was its wide distribution across a large number of fractions, resulting in its reduced amount per fraction. The very low concentration of the substance, distributed across several fractions, resulted in it not being one of the major compounds in terms of concentration in any of these fractions. However, the continued presence of the compound’s peak in the HPTLC densitograms resulted in the incorrect prediction of its contribution to the activity, making it a false positive.

#### 2.4.2. “Blind” Method for the Detection of Compounds with DPPH Scavenging Activity from HPTLC Densitograms

The second approach applied for the detection of bioactive substances via HPTLC was the “blind” method, as reported in Chaita et al. [36]. In that work, the binned densitograms of *Morus alba* FCPC fractions were used for correlation with their activity against mushroom tyrosinase via multivariate analysis, with promising results. Nevertheless, due to lack of this method’s success rate since this was a plant extract, we opted to validate the methodology with an artificial mixture of known composition. In this study, the binned densitograms generated by processing with the rTLC v.1.0 software were used (687 Rf points per sample). For the statistical processing of the results, both multivariate analysis and HetCA studies were performed, with data evaluated for each plate separately. For the multivariate analysis, coefficient plots were created using the SIMCA v14.1.0.2047 software and juxtaposed with the corresponding HPTLC chromatograms (Figure S20), while similar plots were prepared for the HetCA study, via the free DAFdiscovery platform (Figure S21). Option 3 of DAFdiscovery was used, where the NMR data are correlated with the bioactivity. In this study, the data from the binned HPTLC densitograms were processed in an Excel CSV file as NMR data and correlated with the DPPH scavenging activity. Of the two studies, the HetCA study produced better results (nine active compounds and one false positive result were obtained). Nevertheless, the results obtained from the peak integration method were clearly superior. In addition, the presence of several compounds with similar Rf values in the same development system posed a challenge, since the analysis involved multiple fractions simultaneously (17/plate). Considering these factors, the “blind” method was ultimately rejected.

## 3. Materials and Methods

The ArtExtr preparation, FCPC fractionation, evaluation of free radical scavenging activity by DPPH assay, and NMR spectroscopy were all reported by Amountzias et al. [37].

### 3.1. Solvents and Reagents

All solvents were of analytical grade and were purchased from Merck (Merck, Darmstadt, Germany). Water was produced by a LaboStar PRO TWF system (Evoqua Water Technologies, Pittsburgh, PA, USA).

### 3.2. High-Performance Thin Layer Chromatography (HPTLC)

For the chemical profiling of the FCPC fractions of the artificial extract, the filtered samples were re-diluted in methanol at a concentration level of 3 mg/mL. Subsequently, 7 μL of each sample was applied on HPTLC normal phase aluminum plates (20 × 10 cm) precoated with silica gel 60 F_254_ (150–200 mm), and HPTLC reversed aluminum plates (20 × 10 cm) precoated with silica gel 60 RP-18 F_254S_ (150–200 mm) (Merck, Darmstadt, Germany) as 7 mm bands, using an automatic TLC Sampler 4 (ATS-4, CAMAG, Muttenz, Switzerland). The chromatographic separation was performed in the Automatic Developing Chamber 2 (ADC 2) with a solvent system consisting of Tol, EtOAc, and Fa (60/40/1 *v*/*v*/*v*) for the normal phase, and H_2_O, MeCN, and Fa (70/30/1 *v*/*v*/*v*) for the reversed phase, up to a migration distance of 75 mm (from the lower plate edge). The same conditions were used for the development of all the plates. The plates were then documented under 254 nm, 366 nm, and under white light after derivatization with the sulphuric vanillin reagent with CAMAG Visualizer 2. The system was operating under the VisionCats 3.0 software (CAMAG, Muttenz, Switzerland).

### 3.3. Software for Pre- and Post-Processing of HPTLC Chromatograms and Densitograms

The HPTLC chromatogram photos were processed using the free software rTLC v.1.0 [3], which converted the photos into 1000 data points/track that were then exported as Excel CSV files. Of these, only 687 points/track constituted the corresponding densitogram, while the rest were the areas below the baseline and above the solvent front, so they were discarded. The experimental parameters were used to obtain the data (height of sample application, track application length, track distance, distance of the solvent front from the edge of the plate). Finally, all the zones were considered, excluding a total of 1 mm from the outer edges of the spots.

The densitograms were then processed using the free Fityk v. 1.3.1 software [40] to perform baseline correction, which was accomplished through the cubic spline method. In addition, the peaks at the respective wavelengths were integrated using the same software with the GaussianA function, and an integration table was created for statistical processing. Deconvolution of the peaks was applied when necessary.

### 3.4. Nuclear Magnetic Resonance (NMR) Spectroscopy

For the ^1^H-NMR experiment, the samples were dissolved in methanol-*d*_4_ containing tetramethylsilane (TMS) as a reference (Euriso-Top, Saint-Aubin, France) at a concentration level of 10 mg/mL for the unfiltered AF samples and 3 mg/mL for the filtered ArtExtr FCPC samples. After sonication (5 min) in Ultra Sonic bath (Elma Schmidbauer GmbH, Singen, Germany), 650 μL were transferred to 5 mm NMR tubes (LabScape, Bruker, Germany). The ^1^H-NMR spectra were acquired at 298 K, after a 5 min resting period for temperature stabilization, on a Bruker Avance III 600 MHz NMR spectrometer (Bruker, Karlsruhe, Germany) equipped with a 5 mm PABBI 1H/D-BB inverse detection probe. Experiments were performed in automation mode, using a BACS-60 sample changer operated by IconNMR 5.0.7_43. Data acquisition and processing were done with Bruker TopSpin 3.6. Profiling ^1^H-NMR spectra were acquired using water suppression 1D NOESY pulse program (noesygppr1d, Bruker, Karlsruhe, Germany) with the following settings: relaxation delay (d1) = 6 s; acquisition time = 2.73 s; FID (free induction decay) data points = 64k; spectral width = 20 ppm; number of scans = 128. The transmitter offset was set manually in order to achieve optimal suppression of the residual water signal. FIDs were multiplied by an exponential weighting function corresponding to a line broadening of 0.3 Hz prior to Fourier transformation. Automated processing was carried out for phase correction and baseline correction. Chemical shift values were referenced to the residual methanol signal (3.31 ppm).

### 3.5. Methods of Statistical Analysis for the Detection of Bioactive Compounds

#### 3.5.1. Heterocovariance Approach

##### Peak Integration Method

The HetCA study was performed using the free Data Fusion-based Discovery (DAFdiscovery) platform [41]. The Integr. matrix-0 was employed as the X variables, while the DPPH scavenging activity of the fractions was used as the Y variable. Option 4 of DAFdiscovery was used, where the MS data are correlated with the bioactivity. In this study, the data from the integration of the HPTLC peaks were processed in an Excel CSV file as MS data and correlated with the DPPH scavenging activity.

The sHetCA method was based on the correlation and covariance algorithms built into Excel (Microsoft Corporation, Redmond, WA, USA). The Integr. matrix was used to generate a correlation matrix, while covariance was calculated only for the substances that exhibited a positive correlation coefficient with the activity.

##### “Blind” Method

For the “blind” method, option 3 of DAFdiscovery was used, where the NMR data are correlated with the bioactivity. In this study, the data from the binned HPTLC densitograms were processed in an Excel CSV file as NMR data and correlated with the DPPH scavenging activity. This process produced a HetCA plot, where the Y-axis corresponds to the covariance and the X-axis to the Rf value.

#### 3.5.2. Multivariate Analysis

For multivariate data analysis, the Integr. matrix-0 was used as the X variables, while the DPPH scavenging activity of the fractions was employed as the Y variable. Partial least squares (PLS) and orthogonal partial least squares (OPLS) analyses were performed using SIMCA v14.1.0.2047 software (Umetrics, Umeå, Sweden). Unit variance scaling (UV) and Pareto scaling methods were applied for the modeling and regression (CoeffMLR) and correlation (p(corr)) coefficients were calculated. All models were validated using permutation analysis (200 random permutations), whereas the residuals normal probability plot, the Hotelling’s T^2^ Range, the distance to model (DModX) plot, the observed vs. predicted scatterplot, and the Scores vs. CV-Scores scatterplot were examined in order to evaluate the statistical agreement of the proposed model with the experimental one, assess the model’s fitness and robustness and detect any possible outliers. Additionally, CV-ANOVA was performed to investigate the probability that a model could be the result of chance. The Coefficient and VIP plots were used to obtain the results. Similar to the HetCA method, each plot point was additionally color-coded according to the corresponding correlation coefficients (ranging from blue for low correlation to deep red for high correlation). Furthermore, for the “blind” method, data from the binned HPTLC densitograms were imported into an Excel file as X variables and the activity against DPPH of the ArtExtr fractions was defined as the Y variable. Multivariate analysis was performed as previously described.

## 4. Conclusions

In this study, a complex mixture of 59 standard compounds was used as a case study to analyze and compare chemometric methodologies for detecting substances with DPPH activity using the HPTLC technique, following fractionation via FCPC. The two main approaches used were the peak integration method and the “blind” method, in order to evaluate the performance and accuracy of each method. Of the 59 compounds in the ArtExtr, 49 were identified and 43 were integrated. While the “blind” method produced poor results and was therefore rejected, the peak integration method proved significantly more effective. The HetCA method performed via the DAFdiscovery platform was also rejected due to the mandatory usage of the Integr. matrix-0, which produced erroneous results. The sHetCA and the multivariate statistics methods correctly predicted 12 (85.7%) and 13 (92.9%) out of 14 detected active compounds, respectively. Their overall success rates were 63.2% and 68.4%, respectively, out of 19 active compounds included in the study, while the false positive results were 12 and 15, respectively. While both methodologies can be used to explore the bioactive compounds of a mixture, sHetCA produces consistently reliable results with fewer false positive results and without the need for additional diagnostic analyses, making it the preferred approach.

Ensuring a broad distribution of components within the fractions resulting from chromatographic fractionation is vital for effectively applying chemometrics-based methodologies. Profiling the chemical content of these fractions through chromatographic (HPTLC) and spectroscopic (NMR) techniques is appropriate for assessing the distribution of substances. Consistent protocols for sample preparation, HPTLC application, and evaluation of the fractions’ biological properties are essential to minimize procedural influences on the study’s results. While commercial software and instrumentation can produce good results, carefully examining the HPTLC chromatograms is crucial in order to select the optimal software for processing the chromatograms and/or generated densitograms.

False negative results are primarily caused by low compound concentrations, combined with the co-elution of more active compounds at higher concentrations. Conversely, similar distributions of active and inactive substances within the same fractions, along with cumulative or synergistic effects of co-eluted substances and minimal variation in the fractions’ bioactivity, can result in false positive outcomes. Incorrect peak integration can also result in erroneous results. Using different derivatizing reagents to detect diverse compound classes, employing multiple mobile phases and 2D HPTLC for better metabolite separation and peak integration, utilizing HPTLC databases and hyphenating with other techniques, such as mass spectrometry, can reduce erroneous results and increase the success rate of the methodology. The prioritization of the spots predicted as active through the implementation of HPTLC-sHetCA streamlines the isolation process, reducing the laboratory time and organic solvent volume required to study a plant extract.

HPTLC combined with the sHetCA methodology offers a promising alternative approach for the detection of bioactive compounds in complex mixtures when bioautography is not feasible due to cost or limited availability of necessary reagents. Further investigation is needed to address more complex bioactivity essays (e.g., enzymes). This would help maximize the detection of bioactive compounds and minimize the occurrence of false positive and negative results, particularly those stemming from synergistic or antagonistic interactions among the mixture components. The peak integration method uses the integration values of observed HPTLC peaks, allowing both known and new natural secondary metabolites to be employed in the application of the sHetCA method. To further validate the proposed methodology, we plan to implement it in a plant extract in the near future.

## Figures and Tables

**Table 1:** List of abbreviations used.

Abbreviation	Abbreviation Meaning
ArtExtr	Artificial extract
CV-ANOVA	Cross-validation analysis of variance
CV-Scores	Cross-validation Scores
DPPH OPLS Par CoeffMLR	OPLS model with Pareto scaling and study of the MLR Coefficients plot
DPPH OPLS UV VIP	OPLS model with UV scaling and study of the VIP plot
FCPC	Fast centrifugal partition chromatography
FLR	Fluorescence spectrometry
HetCA	Heterocovariance approach
HPTLC	High-performance thin layer chromatography
Integr. Matrix	Integration matrix
Integr. matrix-0	Integration matrix where the missing values were replaced with 0
MS	Mass spectrometry
NMR	Nuclear magnetic resonance spectrometry
NP	Normal phase
OPLS	Orthogonal partial least squares regression
PLS	Partial least squares regression
RMSEE	Root mean square error of estimate
RP	Reverse phase
sHetCA	Sparse heterocovariance approach
STOCSY	Statistical total correlation spectroscopy
UV–Vis	Ultraviolet–Visible spectrometry

**Table 2:** Comparison of correct, false positive, and false negative results predicted by HetCA, sHetCA, and multivariate analysis during the DPPH study.

	DAFdiscovery	sHetCA DPPH	sHetCA DPPH Fr20-70	DPPH PLS Par Coef	DPPH PLS Par VIP	DPPH OPLS Par Coef	DPPH OPLS Par VIP	DPPH PLS UV Coef	DPPH PLS UV VIP	DPPH OPLS UV Coef	DPPH OPLS UV VIP
Compounds included in the study	43	43	37	43	43	43	43	43	43	43	43
Correct	27	29	25	27	31	31	28	26	27	30	27
Correct (%)	62.8	67.4	67.6	62.8	72.1	72.1	65.1	60.5	62.8	69.8	62.8
Active compounds included in the study	14	14	14	14	14	14	14	14	14	14	14
Correctly predicted active compounds	13	12	12	10	7	13	7	11	9	11	13
Correctly predicted active compounds (%)	92.9	85.7	85.7	71.4	50	92.9	50	78.6	64.3	78.6	92.9
False positive results	15	12	10	12	5	11	8	14	11	10	15
False negative results	1	2	2	4	7	1	7	3	5	3	1

**Table 3:** Indices of fitting capacity and predictive ability of models derived from the multivariate analysis of HPTLC peaks for the detection of substances with DPPH scavenging activity.

Index	DPPH PLS Pareto	DPPH OPLS Pareto	DPPH PLS UV	DPPH OPLS UV
R^2^X	0.301	0.725	0.232	0.585
R^2^Y	0.875	0.964	0.893	0.963
Q^2^	0.823	0.918	0.832	0.914
PCs	2	1 + 6 + 0	2	1 + 5 + 0

R^2^X: The fraction of the sum of the squares of the variables indicates the variance of the data. R^2^Y: The fraction of the sum of the squares of the activity indicates its variance. Q^2^: The fraction of total variance in X or Y that can be predicted by a component, as estimated by cross-validation. PCs: Main components.

**Table 4:** Example of covariance and correlation coefficient calculation between the compound **23** (curcumin) and the DPPH scavenging activity of the corresponding fractions.

Calculation	Covariance of 23/DPPH	Correlation Coefficient of 23/DPPH	Prediction
Based on all the fractions	−0.05	−0.01	Inactive
Based on fractions Fr20-24	1.05	0.38	Active

**Table 5:** Correlation coefficient values between the concentrations of false positive results and those of active compounds in the respective fractions, as determined by the sHetCA method and multivariate analysis.

Code	02	03	04 ^a^	26	34	41	46	56 ^a^	57
**05 ^b^**	0.97								
**09 ^b^**									0.80
**12 ^b^**	0.83				0.96				
**15 ^b^**			0.97						
**28 ^c^**							0.99		
**32 ^b^**								0.74	
**43 ^c^**		0.79				0.90	0.65		
**47 ^b^**		0.95							
**55 ^b^**				0.96					
**59 ^c^**	0.99								

^a^ Compounds that were integrated in ^1^H-NMR. ^b^ False positive compounds from both sHetCA and the DPPH OPLS Par CoeffMLR model. ^c^ False positive compounds from the DPPH OPLS Par CoeffMLR model.

## Data Availability

The datasets presented in this article are not readily available because this research is part of an ongoing study. Requests to access the datasets should be directed to Vaios Amountzias.

## Supplementary Materials

The following supporting information can be downloaded at: https://www.mdpi.com/article/10.3390/molecules29246027/s1, Table S1. Standard substances of the ArtExtr (name, code, molecular weight) and their activity against DPPH free radicals; Figure S1. ArtExtr ingredients structures; Figure S2. HPTLC chromatograms of FCPC fractions of the ArtExtr. (a) NP at 254 nm, (b) NP at 366 nm, (c) NP in visible light after derivatization with sulfuric vanillin reagent, (d) RP at 254 nm, (e) RP at 366 nm, and (f) RP in visible light after derivatization with sulfuric vanillin reagent; Figure S3. 1H-NMR spectra of the ArtExtr fractions. The region 3.38–3.31 ppm corresponding to the methanol-d4 peak has been excluded from the spectra for better visualization; Figure S4. % DPPH scavenging activity of all the fractions of the ArtExtr at (a) 200 μg/mL and (b) 75 μg/mL. Inhibition is expressed as mean ± SD (N = 3); Figure S5. NP-HPTLC chromatograms of standard compounds (01–59) (a) at 254 nm (b) at 366 nm, (c) under white light before derivatization, (d) under white light after derivatization with sulfuric vanillin reagent, and (e) at 366 nm after derivatization with sulfuric vanillin reagent; Figure S6. RP-HPTLC chromatograms of standard compounds (01–59) (a) at 254 nm, (b) at 366 nm, (c) under white light after derivatization with sulfuric vanillin reagent, and (d) at 366 nm after derivatization with sulfuric vanillin reagent; Figure S7. Chromatogram of standard resveratrol and comparison with the chromatograms of the ArtExtr fractions Fr38-45 in NP (a) at 254 nm and (b) under white light after derivatization with sulfuric vanillin reagent, and RP (c) at 254 nm and (d) under white light after derivatization with sulfuric vanillin reagent. The spot 06, which corresponds to resveratrol, is highlighted; Table S2. Selected biphasic solvent systems for the FCPC stepwise elution-extrusion fractionation of the ArtExtr; Figure S8. Example of the densitograms of fractions Fr53 and Fr54, in RP at 254 nm, via rTLC software v.1.0. In the red frame, the differences of the three RGB channels, as well as of their average (grayscale) regarding the peak intensity of the blue spot at Rf = 0.2–0.3 are shown. (a) Red channel; (b) green channel; (c) blue channel; and (d) grayscale; Figure S9. (a) Example of baseline correction (red line) in the densitograms of fractions Fr52–70, at 254 nm, RP. The peak that has the opposite sign compared to the rest, at Rf = 0.2–0.3, and which corresponds to the blue spot, is evident; (b) the same densitograms after processing; Table S3. Table with information about the integration of densitogram peaks; Figure S10. Heterocovariance plot of HPTLC peaks integrals with % DPPH scavenging activity by the free DAFdiscovery platform; Table S4. Results of heterocovariance approach, sparse heterocovariance approach, and multivariate analysis for the detection of compounds with DPPH scavenging activity; Figure S11. Permutation analysis with 200 permutations for each model: (a) DPPH PLS Pareto scaling; (b) DPPH OPLS Pareto scaling; (c) DPPH PLS UV scaling; and (d) DPPH OPLS UV scaling; Figure S12. Scatterplots of the predicted versus observed values of the (a) DPPH PLS Par; (b) DPPH OPLS Par; (c) DPPH PLS UV; and (d) DPPH OPLS UV models; Figure S13. Hotelling T2Range plots of the (a) DPPH PLS Par; (b) DPPH OPLS Par; (c) DPPH PLS UV; and (d) DPPH OPLS UV models. The green line shows suspect outliers (95% confidence), while the red line shows strong outliers (99% confidence); Figure S14. DModX plots of the (a) DPPH PLS Par; (b) DPPH OPLS Par; (c) DPPH PLS UV; and (d) DPPH OPLS UV models. Observations with values twice the critical DModX values (red line) are outliers; Figure S15. Normal probability plots of residuals of the (a) DPPH PLS Par; (b) DPPH OPLS Par; (c) DPPH PLS UV; and (d) DPPH OPLS UV models; Figure S16. Scores and cross-validation scores (CV-Scores) plots, respectively, of the (a) DPPH PLS Par; (b) DPPH OPLS Par; (c) DPPH PLS UV; and (d) DPPH OPLS UV models; Figure S17. Coefficient plots of the models (a) DPPH PLS Par; (b) DPPH OPLS Par; (c) DPPH PLS UV; and (d) DPPH OPLS UV; Figure S18. VIP plots of the models (a) DPPH PLS Par; (b) DPPH OPLS Par; (c) DPPH PLS UV; and (d) DPPH OPLS UV; Figure S19. Concentration variance of compounds (a) 09 and 57 (Fr40-48); (b) 02, 12, and 34 (Fr49-54); (c) 28 and 46 (Fr37-46); (d) HPTLC-32 and NMR-56 (Fr26-37); (e) 03, 41, 43, and 46 (Fr35-40); (f) 03 and 47 (Fr36-41); and (g) 26 and 55 (Fr60-66) and % DPPH scavenging activity of respective fractions; Figure S20. HPTLC chromatograms juxtaposed with the corresponding coefficient plots of DPPH scavenging activity of fractions Fr18-34 in normal phase, obtained from the multivariate analysis (a) at 254 nm; (b) at 366 nm; (c) at visible light; and (d) in visible light after derivatization with sulfuric vanillin reagent. Examples of spots that appear to be highly correlated with the activity are listed in a red box; Figure S21. HPTLC chromatograms juxtaposed with the corresponding HetCA plots of DPPH scavenging activity of fractions Fr18-34 in normal phase, obtained from the heterocovariance approach (a) at 254 nm; (b) at 366 nm; (c) at visible light; and (d) in visible light after derivatization with sulfuric vanillin reagent. Examples of spots that appear to be highly correlated with the activity are listed in a red box.
