# Supplementary material for: HPTLC Combined with sHetCA and Multivariate Statistics for the Detection of Bioactive Compounds in Complex Mixtures
Source: Molecules. 2024 Dec 20;29(24):6027. doi: 10.3390/molecules29246027 (PMC11679881; doi:10.3390/molecules29246027)
Supplement: Supplementary file 1 [file molecules-29-06027-s001.zip › molecules-3332503-supplementary.pdf]

molecules

MDPI

Article

Supplementary Materials

HPTLC Combined with sHetCA and Multivariate Statistics for the Detection of Bioactive Compounds in Complex Mixtures

Vaios Amountzias [1,*], Evagelos Gikas [2,*] and Nektarios Aligiannis [1]

[1] Department of Pharmacognosy and Natural Products Chemistry, Faculty of Pharmacy, National and Kapodistrian University of Athens, 15771 Athens, Greece; aligiannis@pharm.uoa.gr
[2] Department of Analytical Chemistry, Faculty of Chemistry, National and Kapodistrian University of Athens, 15771 Athens, Greece
* Correspondence: amountziasv@pharm.uoa.gr (V.A.); vgikas@chem.uoa.gr (E.G.)

Table S1. Standard substances of the ArtExtr (name, code, molecular weight) and their activity against DPPH free radicals.
Table S2. Selected biphasic solvent systems for the FCPC stepwise elution-extrusion fractionation of the ArtExtr.
Table S3. Table with information about the integration of densitogram peaks.
Table S4. Results of Heterocovariance approach, sparse heterocovariance approach and multivariate analysis for the detection of compounds with DPPH scavenging activity.
Figure S1. ArtExtr ingredients structures.
Figure S2. HPTLC chromatograms of FCPC fractions of the ArtExtr. (a) NP at 254 nm, (b) NP at 366 nm, (c) NP in visible light after derivatization with sulfuric vanillin reagent, (d) RP at 254 nm, (e) RP at 366 nm, and (f) RP in visible light after derivatization with sulfuric vanillin reagent.
Figure S3. 1H-NMR spectra of the ArtExtr fractions. The region 3.38-3.31 ppm corresponding to the methanol-d4 peak has been excluded from the spectra for better visualization.
Figure S4. % DPPH scavenging activity of all the fractions of the ArtExtr at (a) 200 μg/mL and (b) 75 μg/mL. Inhibition is expressed as mean ± SD (N=3).
Figure S5. NP-HPTLC chromatograms of standard compounds (01-59) (a) at 254 nm (b) at 366 nm, (c) under white light before derivatization, (d) under white light after derivatization with sulfuric vanillin reagent and (e) at 366 nm after derivatization with sulfuric vanillin reagent.
Figure S6. RP-HPTLC chromatograms of standard compounds (01-59) (a) at 254 nm; (b) at 366 nm, (c) under white light after derivatization with sulfuric vanillin reagent and (d) at 366 nm after derivatization with sulfuric vanillin reagent.
Figure S7. Chromatogram of standard resveratrol and comparison with the chromatograms of the ArtExtr fractions Fr38-45 in NP (a) at 254 nm and (b) under white light after derivatization with sulfuric vanillin reagent, and RP (c) at 254 nm and (d) under white light after derivatization with sulfuric vanillin reagent. The spot 06, which corresponds to resveratrol is highlighted.
Figure S8. Example of the densitograms of fractions Fr53 and Fr54, in RP at 254 nm, via rTLC software v.1.0. In the red frame, the differences of the three RGB channels, as well as of their average (gray scale) regarding the peak intensity of the blue spot at Rf = 0.2-0.3 are shown. (a) Red channel; (b) Green channel; (c) Blue channel and (d) Gray scale.
Figure S9. (a) Example of baseline correction (red line) in the densitograms of fractions Fr52–70, at 254 nm, RP. The peak that has the opposite sign compared to the rest, at Rf = 0.2-0.3 and which corresponds to the blue spot, is evident; (b) The same densitograms after processing.
Figure S10. Heterocovariance plot of HPTLC peaks integrals with % DPPH scavenging activity by the free DAFdiscovery platform.
Figure S11. Permutation analysis with 200 permutations for each model: (a) DPPH PLS Pareto scaling; (b) DPPH OPLS Pareto scaling; (c) DPPH PLS UV scaling and (d) DPPH OPLS UV scaling.
Figure S12. Scatterplots of the predicted versus observed values of the (a) DPPH PLS Par; (b) DPPH OPLS Par; (c) DPPH PLS UV and (d) DPPH OPLS UV models.
Figure S13. Hotelling T2Range plots of the (a) DPPH PLS Par; (b) DPPH OPLS Par; (c) DPPH PLS UV and (d) DPPH OPLS UV models. The green line shows suspect outliers (95% confidence), while the red line shows strong outliers (99% confidence).
Figure S14. DModX plots of the (a) DPPH PLS Par; (b) DPPH OPLS Par; (c) DPPH PLS UV and (d) DPPH OPLS UV models. Observations with values twice the critical DModX values (red line) are outliers.

Figure S15. Normal probability plots of residuals of the (a) DPPH PLS Par; (b) DPPH OPLS Par; (c) DPPH PLS UV and (d) DPPH OPLS UV models.
Figure S16. Scores and cross-validation Scores (CV-Scores) plots, respectively, of the (a) DPPH PLS Par; (b) DPPH OPLS Par; (c) DPPH PLS UV and (d) DPPH OPLS UV models.
Figure S17. Coefficient plots of the models (a) DPPH PLS Par; (b) DPPH OPLS Par; (c) DPPH PLS UV and (d) DPPH OPLS UV.
Figure S18. VIP plots of the models (a) DPPH PLS Par; (b) DPPH OPLS Par; (c) DPPH PLS UV and (d) DPPH OPLS UV.
Figure S19. Concentration variance of compounds (a) 09 and 57 (Fr40-48); (b) 02, 12 and 34 (Fr49-54); (c) 28 and 46 (Fr37-46); (d) HPTLC-32 and NMR-56 (Fr26-37); (e) 03, 41, 43 and 46 (Fr35-40); (f) 03 and 47 (Fr36-41) and (g) 26 and 55 (Fr60-66) and % DPPH scavenging activity of respective fractions.
Figure S20. HPTLC chromatograms juxtaposed with the corresponding coefficient plots of DPPH scavenging activity of fractions Fr18-34 in normal phase, obtained from the multivariate analysis (a) at 254 nm; (b) at 366 nm; (c) at visible light and (d) in visible light after derivatization with sulfuric vanillin reagent. Examples of spots that appear to be highly correlated with the activity are listed in a red box.
Figure S21. HPTLC chromatograms juxtaposed with the corresponding HetCA plots of DPPH scavenging activity of fractions Fr18-34 in normal phase, obtained from the heterocovariance approach (a) at 254 nm; (b) at 366 nm; (c) at visible light and (d) in visible light after derivatization with sulfuric vanillin reagent. Examples of spots that appear to be highly correlated with the ac-tivity are listed in a red box.

Table S1. Standard substances of the ArtExtr (name, code, molecular weight) and their activity against DPPH free radicals.

Code	Name	Chemical category	Molecular Weight (g/mol)	mmoles	Molecular fraction (%) in the ArtExtr	DPPH scavenging activity (100 μg/mL)	IC_{50}* DPPH (μM)
01	Galanthamine hydrobromide	Alkaloid	368.30	0.14	1.13	1.2 ± 0.4	
02	Quercitrin	Flavonoid	448.40	0.11	0.89	91.4 ± 0.2	50.5
03	Quercetin	Flavonoid	302.23	0.17	1.37	96.6 ± 0.1	30.3
04	Kaempferol	Flavonoid	286.24	0.17	1.37	96.7 ± 0.0	68.1
05	Phlorizin	Chalcone	436.40	0.11	0.89	0 ± 0.6	
06	Resveratrol	Stilbenoid	228.24	0.22	1.78	74.5 ± 0.7	225.4
07	Aristolochic acid	Monocarboxylic acid	341.27	0.15	1.21	0.0 ± 1.1	
08	Palmitic acid	Fatty acid	256.42	0.19	1.53	0.0 ± 1.2	
09	Reserpine	Alkaloid	608.70	0.08	0.65	0.0 ± 1.0	
10	Caffeic acid	Phenolic acid	180.16	0.28	2.26	95.8 ± 0.1	44.7
11	Rosmarinic acid	Phenolic acid	360.30	0.14	1.13	95.8 ± 0.0	37.7
12	Ephedrine	Alkaloid	165.23	0.30	2.42	0.0 ± 0.4	
13	Harmine	Alkaloid	212.25	0.24	1.94	0.0 ± 0.8	
14	Oleanolic acid	Terpenoid	456.70	0.11	0.89	0.0 ± 0.4	
15	Naringenin	Flavonoid	272.25	0.18	1.45	0.0 ± 0.4	
16	Hesperetin	Flavonoid	302.28	0.17	1.37	40.2 ± 0.4	
17	Nicotinic acid	Pyridinecarboxylic acid	123.11	0.41	3.31	0.4 ± 0.5	
18	Shikonin	Naphthoquinone	288.29	0.17	1.37	41.0 ± 3.1	
19	Thymol	Phenol	150.22	0.33	2.66	18.6 ± 0.6	
20	Oxytetracycline hydrochloride	Tetracycline (Alkaloid)	496.90	0.10	0.81	8.0 ± 0.5	
21	18-β-glycyrrhetinic acid	Terpenoid	470.70	0.11	0.89	0.0 ± 1.6	
22	2,4-dimethoxyphenylacetic acid	Phenolic acid	196.20	0.25	2.02	0.0 ± 0.4	

23	Curcumin	Polyphenol	368.40	0.14	1.13	95.6 ± 0.2	80.8
24	Oleuropein	Iridoid	540.50	0.09	0.73	96.2 ± 0.0	61.1
25	Sucrose	Sugar	342.30	0.15	1.21	0.0 ± 0.7	
26	Rutin	Flavonoid	610.50	0.08	0.65	91.6 ± 0.0	46.8
27	Arbutin	Phenol	272.25	0.18	1.45	41.1 ± 0.5	
28	*p*-Coumaric acid	Phenolic acid	164.16	0.30	2.42	1.4 ± 0.5	
29	Homovanillic acid	Phenolic acid	182.17	0.27	2.18	66.5 ± 0.3	261.3
30	Ellagic acid (dihydrate)	Polyphenol	338.22	0.15	1.21	95.7 ± 0.0	20.8
31	Tannic acid	Polyphenol	1701.20	0.03	0.24	96.4 ± 0.1	3.1
32	Caffeine	Xanthine	194.19	0.26	2.10	0.0 ± 0.8	
33	Vanillin	Benzaldehyde	152.15	0.33	2.66	2.4 ± 0.5	
34	Gallic acid	Phenolic acid	170.12	0.29	2.34	95.7 ± 0.0	30.2
35	Biochanin A	Flavonoid	284.26	0.18	1.45	0.0 ± 0.4	
36	Daidzein	Flavonoid	254.24	0.20	1.61	0.0 ± 0.3	
37	Naringin	Flavonoid	580.50	0.09	0.73	0.0 ± 1.3	
38	Catechol	Phenol	110.11	0.45	3.63	95.9 ± 0.1	48.2
39	3,5-dihydroxybenzoic acid	Phenolic acid	154.12	0.32	2.58	1.0 ± 0.3	
40	D-(−)-quinic acid	Cyclitol	192.17	0.26	2.10	0.0 ± 0.9	
41	Ferulic acid	Phenolic acid	194.18	0.26	2.10	90.2 ± 0.3	95.2
42	4-hydroxybenzoic acid	Phenolic acid	138.12	0.36	2.91	0.0 ± 0.6	
43	*m*-Coumaric acid	Phenolic acid	164.16	0.30	2.42	2.2 ± 2.3	
44	Isoferulic acid	Phenolic acid	194.18	0.26	2.10	22.4 ± 0.1	
45	4-Hydroxybenzaldehyde	Benzaldehyde	122.12	0.41	3.31	0.0 ± 1.2	
46	Sinapic acid	Phenolic acid	224.21	0.22	1.78	93.5 ± 0.1	82.2
47	Vanillic acid	Phenolic acid	168.15	0.30	2.42	6.1 ± 0.1	
48	Diosgenin	Terpenoid	414.60	0.12	0.97	0.0 ± 1.0	
49	*p*-Hydroxyphenylacetic acid	Phenolic acid	152.15	0.33	2.66	0.0 ± 0.7	
50	Chlorogenic acid	Cyclitol	354.31	0.14	1.13	91.7 ± 0.5	87.9
51	Aucuboside	Iridoid	346.33	0.14	1.13	0.0 ± 0.9	

52	Sclareol	Terpenoid	308.50	0.16	1.29	0.0 ± 0.0	
53	Protocatechic acid	Phenolic acid	154.12	0.32	2.58	92.7 ± 0.3	110.6
54	(−)-Scopolamine methyl bromide	Alkaloid	398.30	0.13	1.05	0.0 ± 0.6	
55	Loganin	Iridoid	390.40	0.13	1.05	0.0 ± 0.5	
56	Baicalein	Flavonoid	270.24	0.19	1.53	95.4 ± 0.0	26.3
57	6,7-Dihydroxycoumarin (Esculetin)	Coumarin	178.14	0.28	2.26	95.2 ± 0.0	27.8
58	Umbelliferone	Coumarin	162.14	0.31	2.50	0.0 ± 0.4	
59	Colchicine	Alkaloid	399.40	0.13	1.05	0.0 ± 0.3	

*IC_{50}: half-maximal inhibitory concentration. Results are expressed as the mean ±SD of three independent experiments.

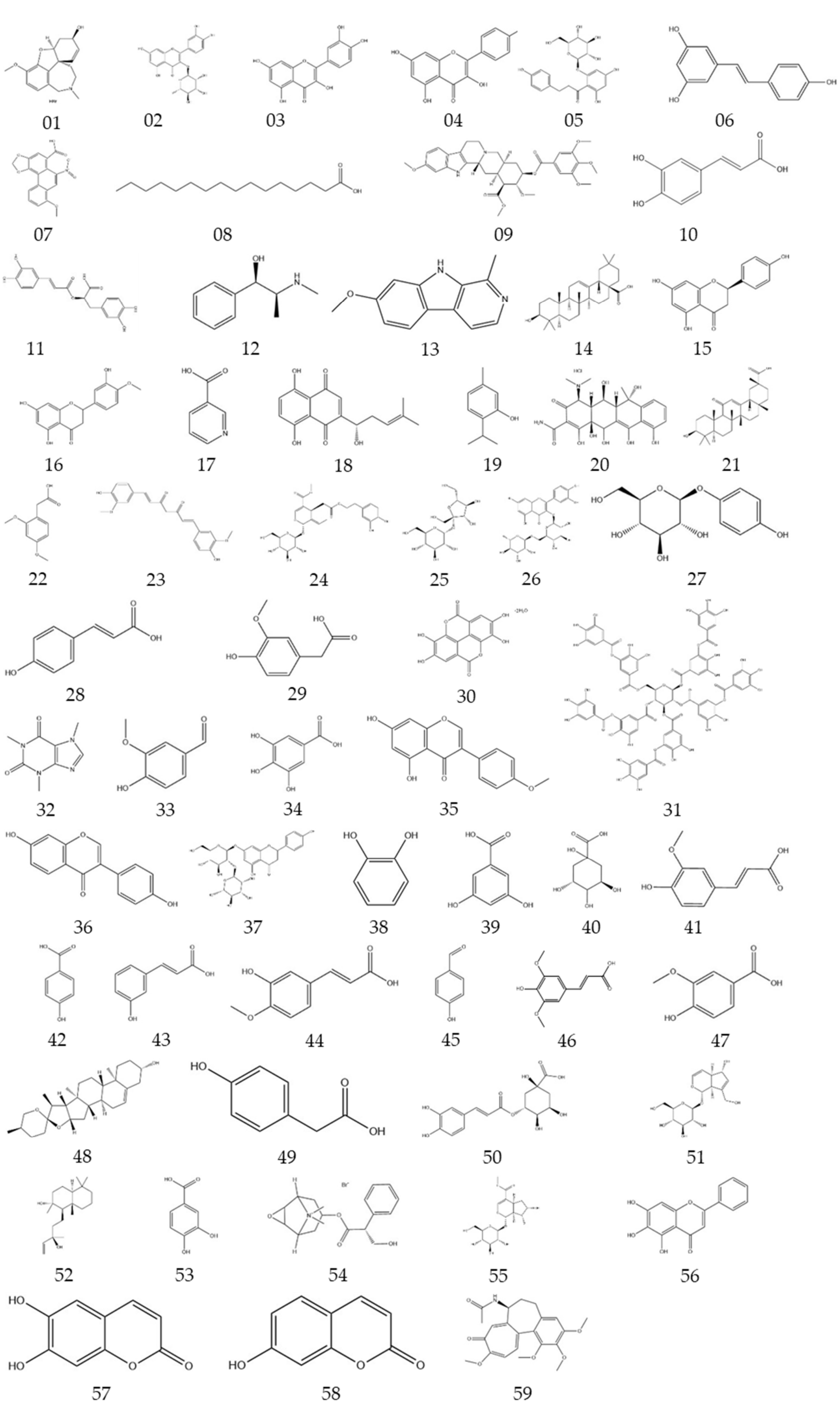

Figure S1. ArtExtr ingredients structures.

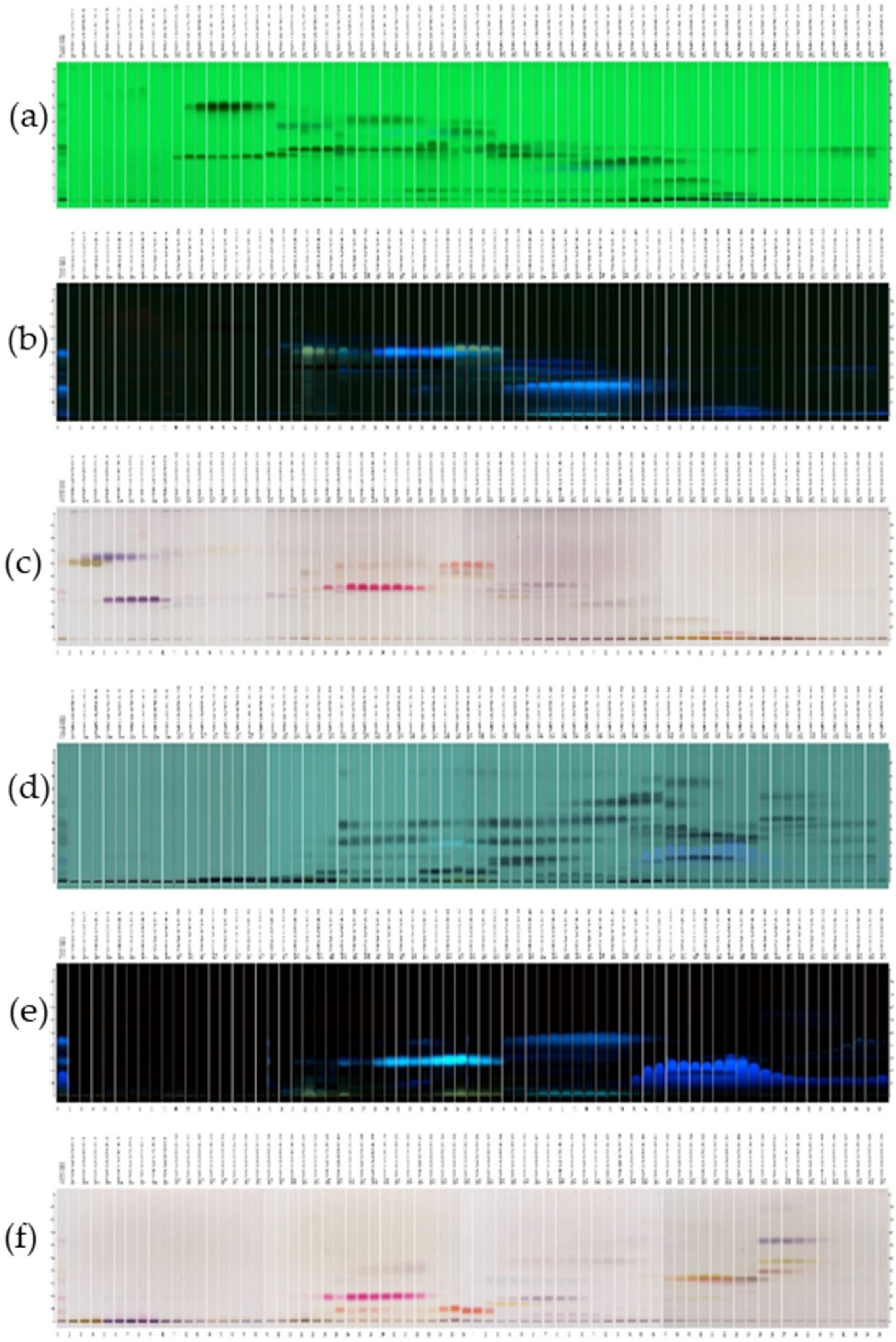

Figure S2. HPTLC chromatograms of FCPC fractions of the ArtExtr. (a) NP at 254 nm, (b) NP at 366 nm, (c) NP in visible light after derivatization with sulfuric vanillin reagent, (d) RP at 254 nm, (e) RP at 366 nm, and (f) RP in visible light after derivatization with sulfuric vanillin reagent.

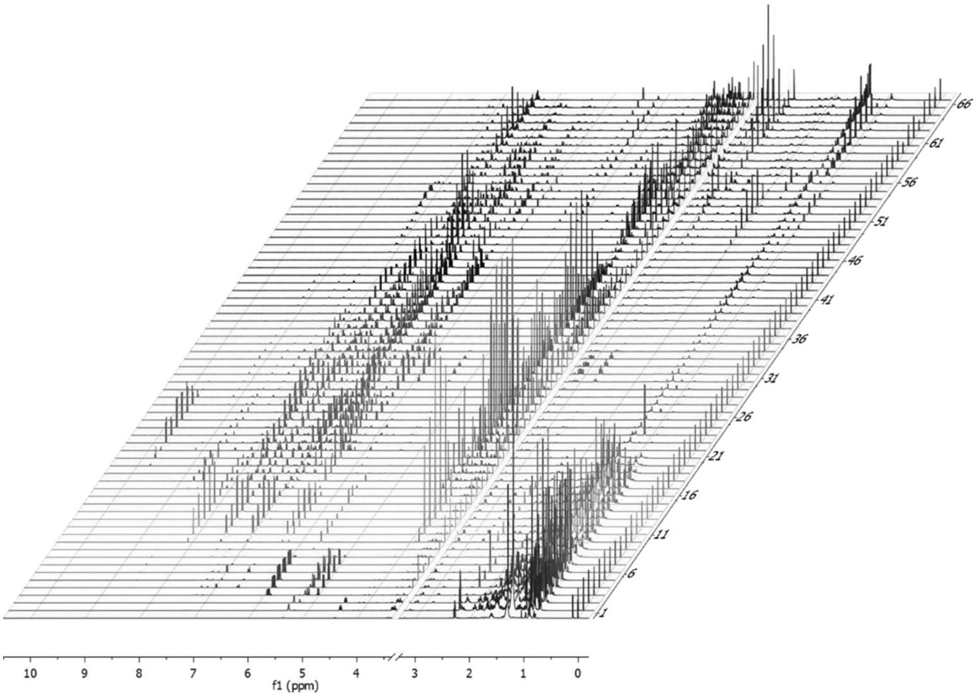

Figure S3. ^{1}H-NMR spectra of the ArtExtr fractions. The region 3.38-3.31 ppm corresponding to the methanol-d_4 peak has been excluded from the spectra for better visualization.

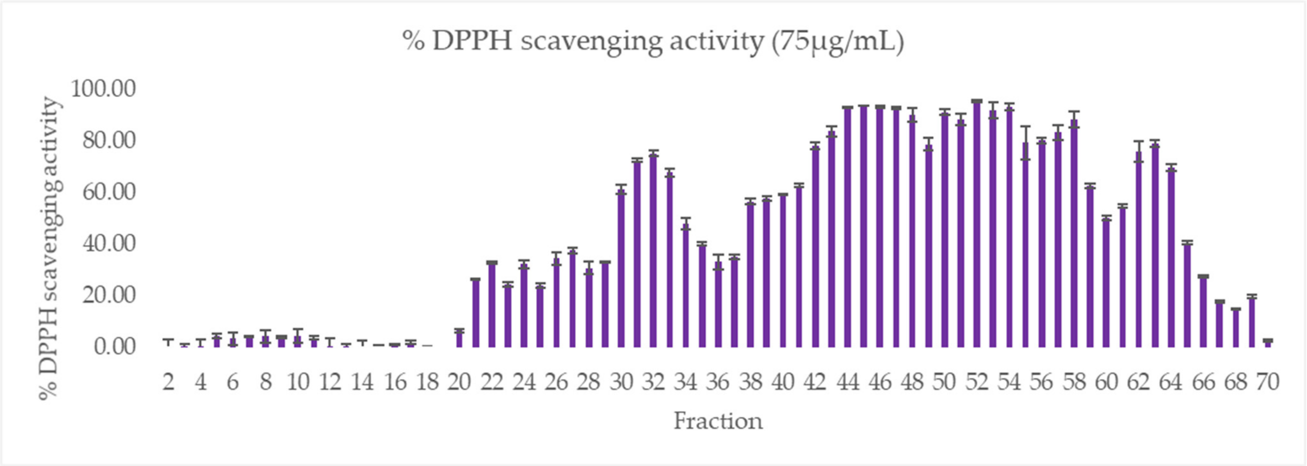

Figure S4. % DPPH scavenging activity of all the fractions of the ArtExtr at (a) 200 µg/mL and (b) 75 µg/mL. Inhibition is expressed as mean ± SD (N=3).

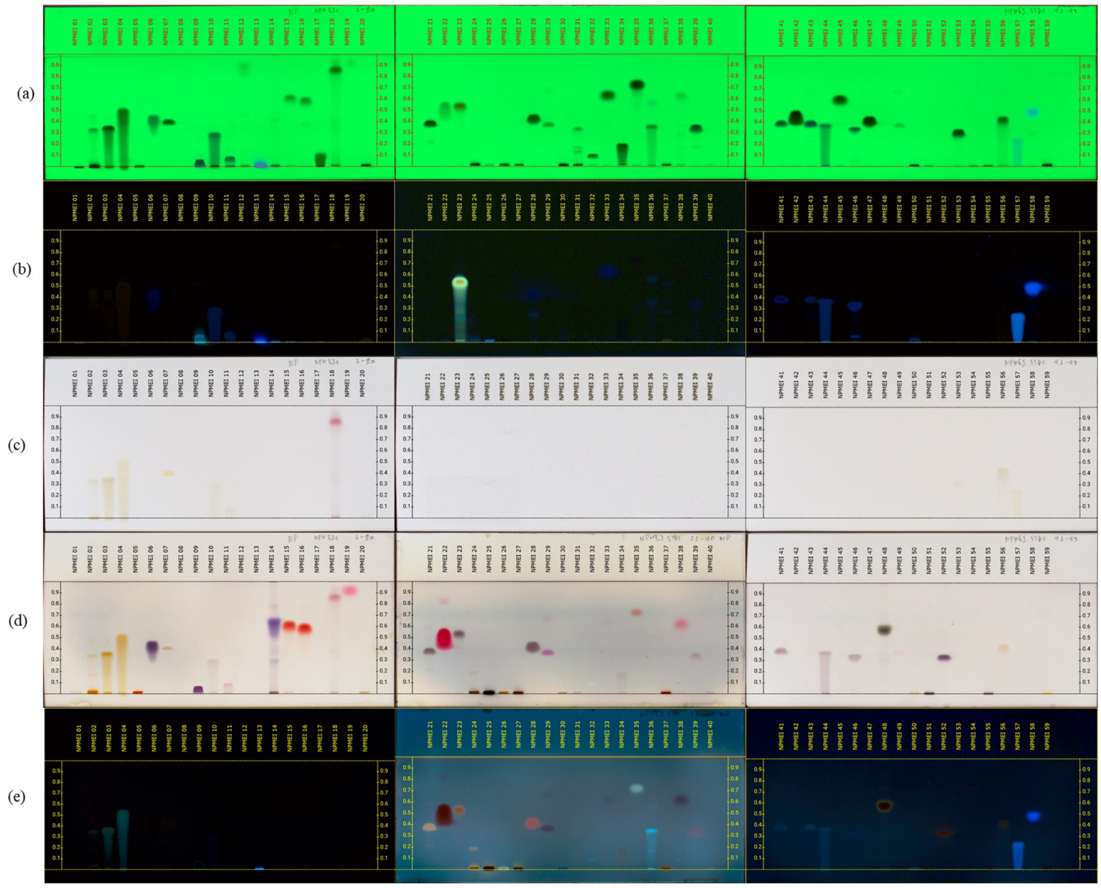

Figure S5. NP-HPTLC chromatograms of standard compounds (01-59) (a) at 254 nm (b) at 366 nm, (c) under white light before derivatization, (d) under white light after derivatization with sulfuric vanillin reagent and (e) at 366 nm after derivatization with sulfuric vanillin reagent.

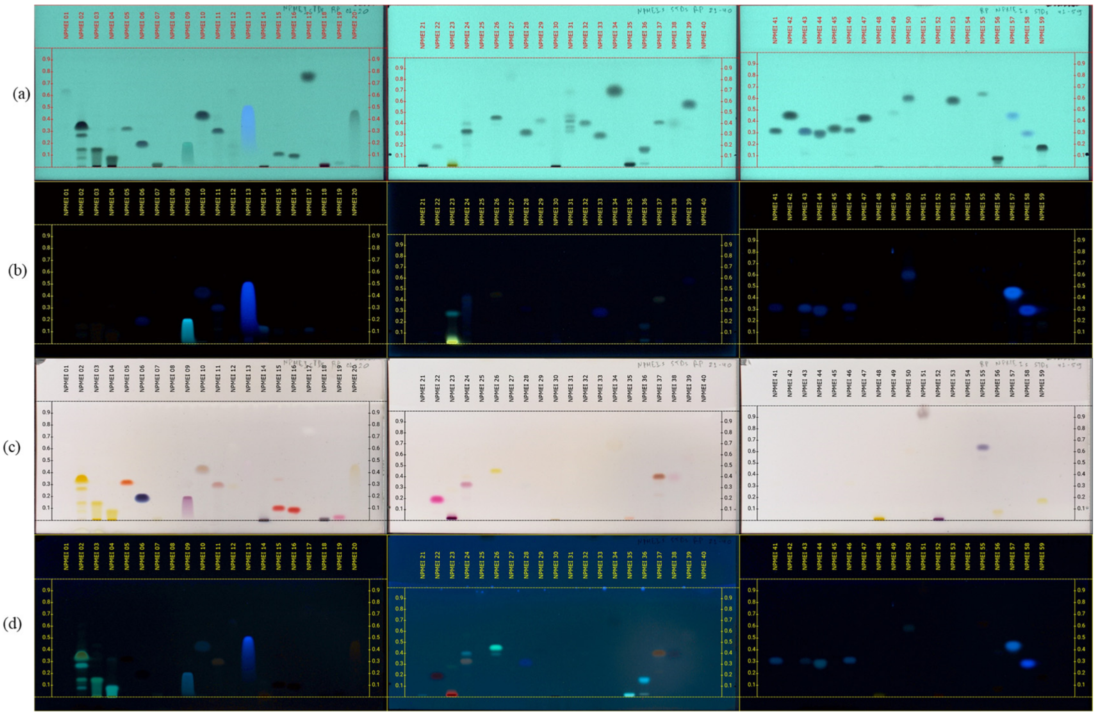

Figure S6. RP-HPTLC chromatograms of standard compounds (01-59) (a) at 254 nm; (b) at 366 nm, (c) under white light after derivatization with sulfuric vanillin reagent and (d) at 366 nm after derivatization with sulfuric vanillin reagent.

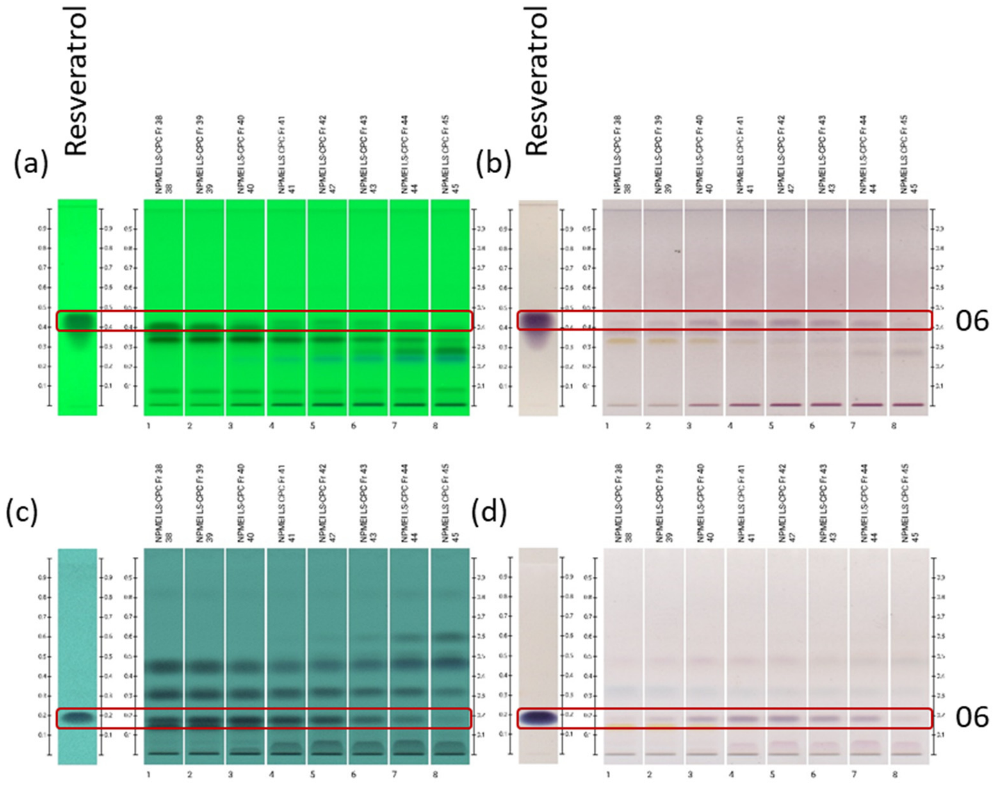

Figure S7. Chromatogram of standard resveratrol and comparison with the chromatograms of the ArtExtr fractions Fr38-45 in NP (a) at 254 nm and (b) under white light after derivatization with sulfuric vanillin reagent, and RP (c) at 254 nm and (d) under white light after derivatization with sulfuric vanillin reagent. The spot 06, which corresponds to resveratrol is highlighted.

Table S2. Selected biphasic solvent systems for the FCPC stepwise elution-extrusion fractionation of the ArtExtr.

	n-Hept	EtOAc	n-BuOH	MeOH	H_2O
S1	9	1	0	5	5
S2	8	2	0	5	5
S3	7	3	0	5	5
S4	6	4	0	5	5
S5	5	5	0	5	5
S6	2	8	0	5	5
S7	2	8	1	4	5
S8	2	8	2	3	5

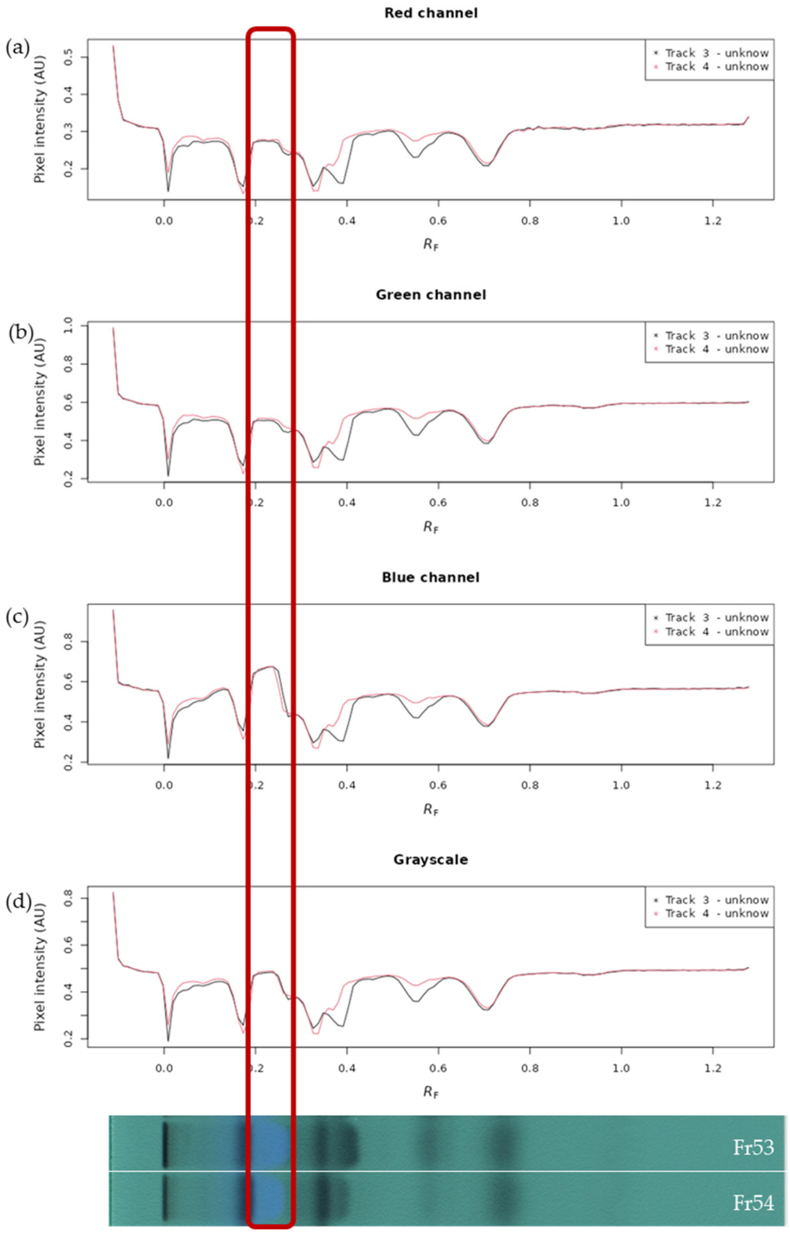

Figure S8. Example of the densitograms of fractions Fr53 and Fr54, in RP at 254 nm, via rTLC software v.1.0. In the red frame, the differences of the three RGB channels, as well as of their average (gray scale) regarding the peak intensity of the blue spot at Rf = 0.2-0.3 are shown. (a) Red channel; (b) Green channel; (c) Blue channel and (d) Gray scale.

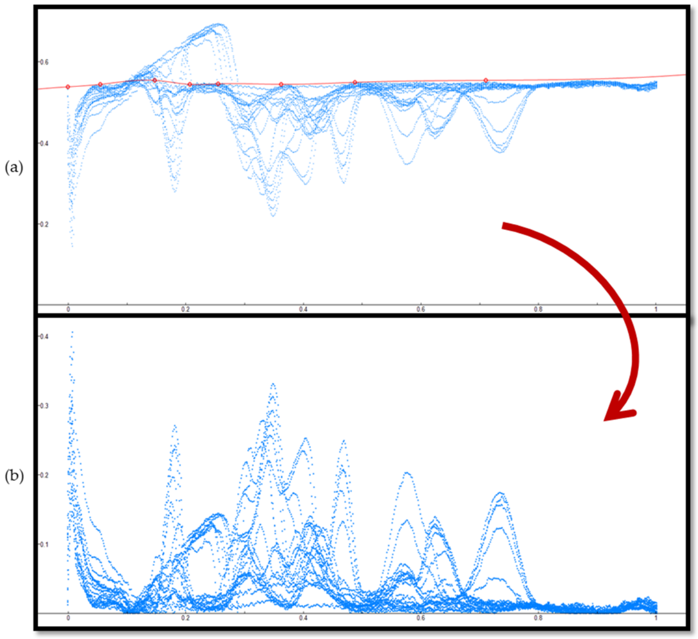

Figure S9. (a) Example of baseline correction (red line) in the densitograms of fractions Fr52–70, at 254 nm, RP. The peak that has the opposite sign compared to the rest, at Rf = 0.2-0.3 and which corresponds to the blue spot, is evident; (b) The same densitograms after processing.

Table S3. Table with information about the integration of densitogram peaks.

Code	Phase	λ (nm)	Rf	Detected	Integrated	Comments
01				No	No	It was not detected
02	RP	254	0.34 to 0.36	Yes	Yes	
03	RP	Van	0.16	Yes	Yes	
04				Yes	No	Unable to integrate due to overlapping
05	RP	366	0.35	Yes	Yes	
06	NP	Van	0.43	Yes	Yes	
07	NP	W	0.39	Yes	Yes	
08				No	No	Cannot be detected with the specific reagents
09	RP	366	0.02	Yes	Yes	
10				No	No	It was not detected
11	NP	Van	0.05	Yes	Yes	
12	RP	Van	0.27	Yes	Yes	
13	RP	254	0.1 to 0.3	Yes	Yes	
14	NP	Van	0.65	Yes	Yes	
15	RP	Van	0.09	Yes	Yes	

16	NP	Van	0.57	Yes	Yes	
17	RP	254	0.82	Yes	Yes	
18	NP	W	0.81	Yes	Yes	
19				No	No	It was not detected
20				No	No	It was not detected
21	NP	254	0.36	Yes	Yes	
22	RP	Van	0.2	Yes	Yes	
23	NP	W	0.52	Yes	Yes	
24	RP	Van	0.32	Yes	Yes	
25				Yes	No	Unable to integrate due to Rf = 1.0
26	RP	254	0.48	Yes	Yes	
27	RP	Van	0.89	Yes	Yes	
28	RP	Van	0.32	Yes	Yes	
29				No	No	It was not detected
30				Yes	No	Unable to integrate due to Rf = 0.0
31				No	No	It was not detected
32	NP	366	0.1	Yes	Yes	
33	RP	254	0.29	Yes	Yes	
34	RP	254	0.76	Yes	Yes	
35	NP	254	0.7	Yes	Yes	
36	NP	254	0.55	Yes	Yes	
37	RP	Van	0.39	Yes	Yes	
38	RP	Van	0.4	Yes	Yes	
39				No	No	It was not detected.
40				No	No	It was not detected
41	NP	Van	0.39	Yes	Yes	
42	RP	254	0.44	Yes	Yes	
43	NP	254	0.37	Yes	Yes	
44				Yes	No	Unable to integrate due to overlapping
45	RP	254	0.32	Yes	Yes	

46	RP	254	0.31	Yes	Yes	
47	RP	254	0.45	Yes	Yes	
48	NP	Van	0.58	Yes	Yes	
49				No	No	It was not detected
50	RP	366	0.58	Yes	Yes	
51				Yes	No	Unable to integrate due to Rf = 1.0
52	NP	Van	0.32	Yes	Yes	
53	RP	254	0.59	Yes	Yes	
54	RP	254	0.31	Yes	Yes	
55	RP	Van	0.61	Yes	Yes	
56				Yes	No	Unable to integrate due to overlapping
57	RP	366	0.44	Yes	Yes	
58	RP	366	0.27	Yes	Yes	
59	RP	Van	0.15 to 0.18	Yes	Yes	

λ: wavelength used, Van: white light after derivatization with sulfuric vanillin reagent, W: white light before derivatization.

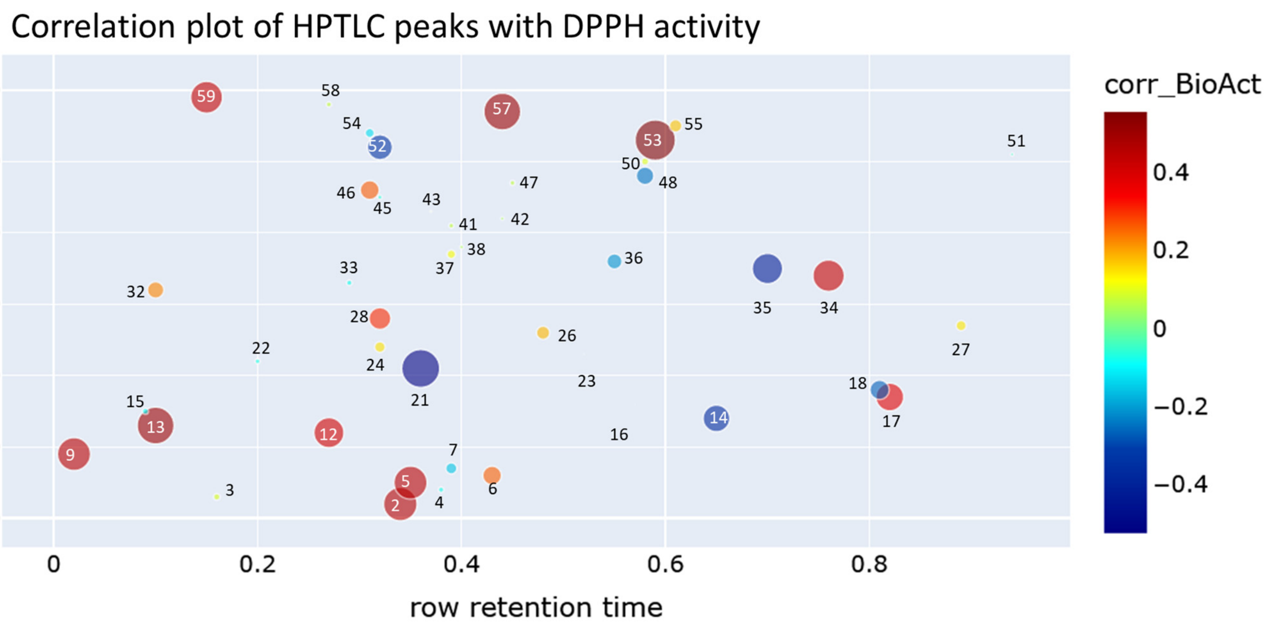

Figure S10. Heterocovariance plot of HPTLC peaks integrals with % DPPH scavenging activity by the free DAFdiscovery platform.

Table S4. Results of Heterocovariance approach, sparse heterocovariance approach and multivariate analysis for the detection of compounds with DPPH scavenging activity.

**Code**	**DAFdiscovery**	**sHetCA DPPH**	**sHetCA DPPH Fr20-70**	**DPPH PLS Par Coef**	**DPPH PLS Par VIP**	**DPPH OPLS Par Coef**	**DPPH OPLS Par VIP**	**DPPH PLS UV Coef**	**DPPH PLS UV VIP**	**DPPH OPLS UV Coef**	**DPPH OPLS UV VIP**
01	N.D.	N.D.	N.D.	N.D.	N.D.	N.D.	N.D.	N.D.	N.D.	N.D.	N.D.
02	Correct	False Negative	False Negative	Correct	Correct	Correct	Correct	Correct	Correct	Correct	Correct
03	Correct	Correct	Correct	Correct	False Negative	Correct	False Negative	Correct	False Negative	Correct	Correct
04	N.D.	N.D.	N.D.	N.D.	N.D.	N.D.	N.D.	N.D.	N.D.	N.D.	N.D.
05	False Positive	False Positive	False Positive	False Positive	False Positive	False Positive	False Positive	False Positive	False Positive	False Positive	False Positive
06	Correct	Correct	Correct	Correct	Correct	False Negative	False Negative	Correct	Correct	False Negative	Correct
07	Correct	False Positive	False Positive	Correct	Correct	Correct	Correct	Correct	Correct	Correct	Correct
08	N.D.	N.D.	N.D.	N.D.	N.D.	N.D.	N.D.	N.D.	N.D.	N.D.	N.D.
09	False Positive	False Positive	False Positive	False Positive	False Positive	False Positive	False Positive	False Positive	False Positive	False Positive	False Positive
10	N.D.	N.D.	N.D.	N.D.	N.D.	N.D.	N.D.	N.D.	N.D.	N.D.	N.D.
11	Correct	Correct	Correct	Correct	Correct	Correct	Correct	Correct	Correct	Correct	Correct
12	False Positive	False Positive	False Positive	False Positive	Correct	False Positive	Correct	False Positive	False Positive	False Positive	False Positive
13	False Positive	False Positive	False Positive	False Positive	False Positive	False Positive	False Positive	False Positive	False Positive	False Positive	False Positive
14	Correct	Correct	n.i.	Correct	Correct	Correct	Correct	Correct	Correct	Correct	Correct
15	Correct	False Positive	False Positive	Correct	Correct	Correct	Correct	Correct	Correct	Correct	Correct
16	Correct	Correct	Correct	Correct	Correct	Correct	Correct	Correct	Correct	Correct	Correct
17	False Positive	Correct	Correct	False Positive	False Positive	False Positive	False Positive	False Positive	False Positive	False Positive	False Positive
18	Correct	False Positive	n.i.	Correct	Correct	Correct	Correct	Correct	Correct	Correct	Correct
19	N.D.	N.D.	N.D.	N.D.	N.D.	N.D.	N.D.	N.D.	N.D.	N.D.	N.D.
20	N.D.	N.D.	N.D.	N.D.	N.D.	N.D.	N.D.	N.D.	N.D.	N.D.	N.D.
21	Correct	Correct	n.i.	Correct	Correct	Correct	Correct	Correct	Correct	Correct	Correct
22	Correct	Correct	Correct	Correct	Correct	Correct	Correct	Correct	Correct	Correct	Correct
23	False Negative	Correct	Correct	False Negative	False Negative	Correct	False Negative	False Negative	False Negative	False Negative	False Negative

24	Correct	Correct	Correct	Correct	False Negative	Correct	False Negative	Correct	False Negative	Correct	Correct
25	N.D.	N.D.	N.D.	N.D.	N.D.	N.D.	N.D.	N.D.	N.D.	N.D.	N.D.
26	Correct	Correct	Correct	False Negative	False Negative	Correct	Correct	Correct	Correct	Correct	Correct
27	False Positive	Correct	Correct	Correct	Correct	Correct	Correct	False Positive	Correct	Correct	False Positive
28	False Positive	Correct	Correct	False Positive	Correct	False Positive	Correct	False Positive	False Positive	False Positive	False Positive
29	N.D.	N.D.	N.D.	N.D.	N.D.	N.D.	N.D.	N.D.	N.D.	N.D.	N.D.
30	N.D.	N.D.	N.D.	N.D.	N.D.	N.D.	N.D.	N.D.	N.D.	N.D.	N.D.
31	N.D.	N.D.	N.D.	N.D.	N.D.	N.D.	N.D.	N.D.	N.D.	N.D.	N.D.
32	False Positive	False Positive	False Positive	False Positive	Correct	False Positive	False Positive	False Positive	False Positive	False Positive	False Positive
33	Correct	Correct	Correct	Correct	Correct	Correct	Correct	Correct	Correct	Correct	Correct
34	Correct	Correct	Correct	Correct	Correct	Correct	Correct	Correct	Correct	Correct	Correct
35	Correct	Correct	n.i.	Correct	Correct	Correct	Correct	Correct	Correct	Correct	Correct
36	Correct	Correct	Correct	Correct	Correct	Correct	Correct	Correct	Correct	Correct	Correct
37	False Positive	Correct	Correct	Correct	Correct	Correct	Correct	False Positive	Correct	Correct	False Positive
38	Correct	Correct	Correct	False Negative	False Negative	Correct	False Negative	False Negative	False Negative	Correct	Correct
39	N.D.	N.D.	N.D.	N.D.	N.D.	N.D.	N.D.	N.D.	N.D.	N.D.	N.D.
40	N.D.	N.D.	N.D.	N.D.	N.D.	N.D.	N.D.	N.D.	N.D.	N.D.	N.D.
41	Correct	Correct	Correct	Correct	False Negative	Correct	False Negative	Correct	Correct	False Negative	Correct
42	False Positive	Correct	Correct	False Positive	Correct	Correct	Correct	False Positive	False Positive	Correct	False Positive
43	False Positive	Correct	Correct	False Positive	Correct	False Positive	False Positive	False Positive	Correct	Correct	False Positive
44	N.D.	N.D.	N.D.	N.D.	N.D.	N.D.	N.D.	N.D.	N.D.	N.D.	N.D.
45	Correct	Correct	Correct	Correct	Correct	Correct	Correct	Correct	Correct	Correct	Correct
46	Correct	False Negative	False Negative	Correct	Correct	Correct	Correct	Correct	Correct	Correct	Correct
47	False Positive	False Positive	False Positive	False Positive	Correct	Correct	Correct	False Positive	False Positive	Correct	False Positive
48	Correct	Correct	n.i.	Correct	Correct	Correct	Correct	Correct	Correct	Correct	Correct
49	N.D.	N.D.	N.D.	N.D.	N.D.	N.D.	N.D.	N.D.	N.D.	N.D.	N.D.
50	Correct	Correct	Correct	False Negative	False Negative	Correct	False Negative	False Negative	False Negative	Correct	Correct

51	N.D.	N.D.	N.D.	N.D.	N.D.	N.D.	N.D.	N.D.	N.D.	N.D.	N.D.
52	Correct	False Positive	n.i.	Correct	Correct	Correct	Correct	Correct	Correct	Correct	Correct
53	Correct	Correct	Correct	Correct	Correct	Correct	Correct	Correct	Correct	Correct	Correct
54	Correct	Correct	Correct	Correct	Correct	Correct	Correct	Correct	Correct	Correct	Correct
55	False Positive	False Positive	False Positive	Correct	Correct	False Positive	False Positive	False Positive	False Positive	False Positive	False Positive
56	N.D.	N.D.	N.D.	N.D.	N.D.	N.D.	N.D.	N.D.	N.D.	N.D.	N.D.
57	Correct	Correct	Correct	Correct	Correct	Correct	Correct	Correct	Correct	Correct	Correct
58	False Positive	False Positive	False Positive	False Positive	False Positive	False Positive	False Positive	Correct	Correct	False Positive	False Positive
59	False Positive	Correct	Correct	False Positive	False Positive	False Positive	False Positive	False Positive	False Positive	False Positive	False Positive

N.D.: Not detected in HPTLC. n.i: Not included in the specific study due to exclusion of fractions Fr02-19.

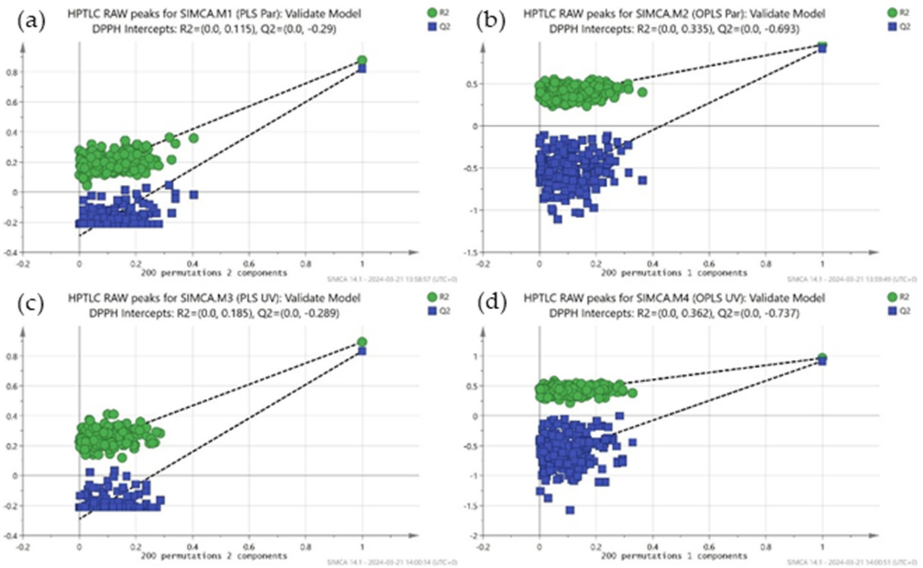

Figure S11. Permutation analysis with 200 permutations for each model: (a) DPPH PLS Pareto scaling; (b) DPPH OPLS Pareto scaling; (c) DPPH PLS UV scaling and (d) DPPH OPLS UV scaling.

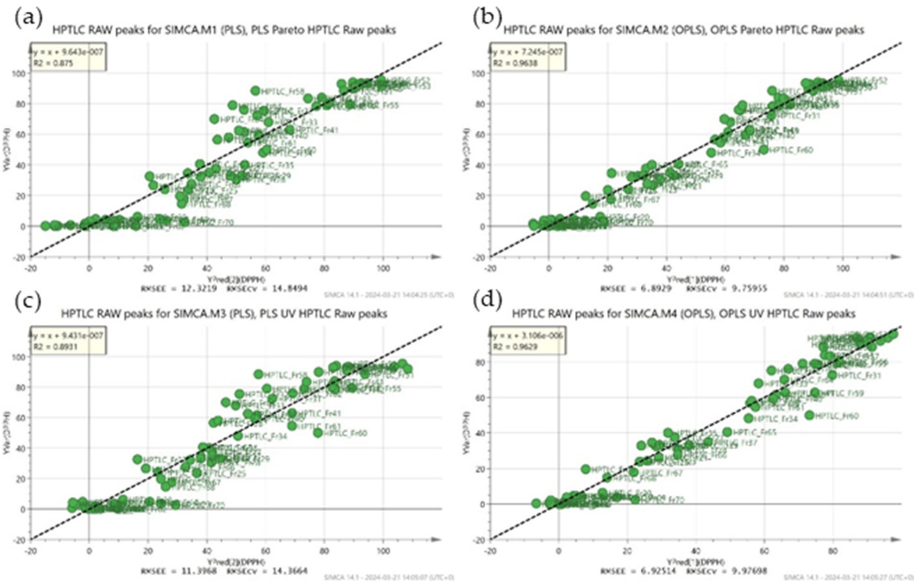

Figure S12. Scatterplots of the predicted versus observed values of the (a) DPPH PLS Par; (b) DPPH OPLS Par; (c) DPPH PLS UV and (d) DPPH OPLS UV models.

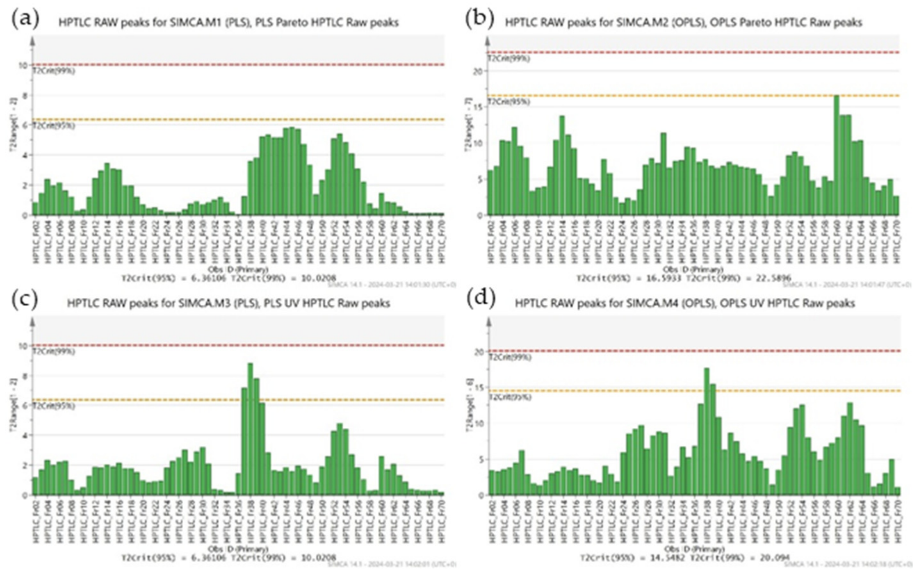

Figure S13. Hotelling T^2Range plots of the (a) DPPH PLS Par; (b) DPPH OPLS Par; (c) DPPH PLS UV and (d) DPPH OPLS UV models. The green line shows suspect outliers (95% confidence), while the red line shows strong outliers (99% confidence).

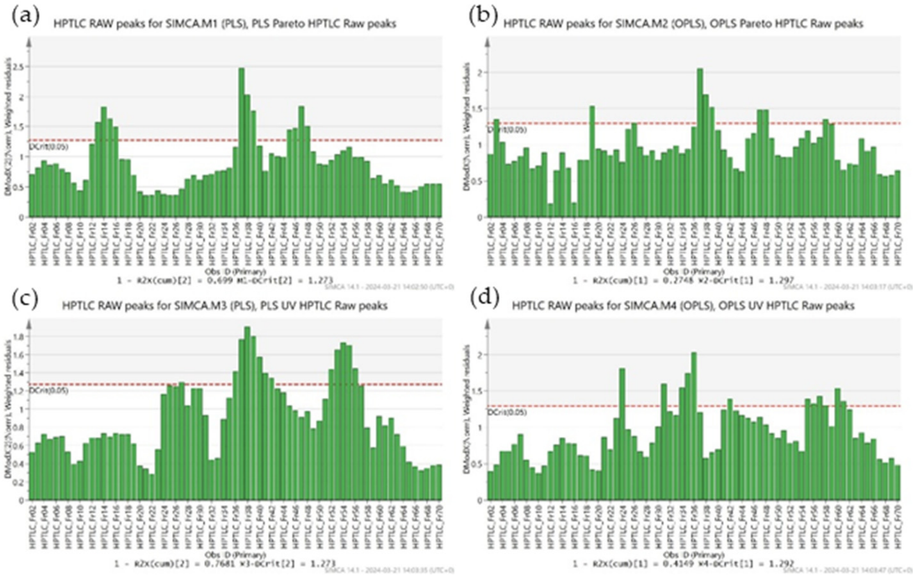

Figure S14. DModX plots of the (a) DPPH PLS Par; (b) DPPH OPLS Par; (c) DPPH PLS UV and (d) DPPH OPLS UV models. Observations with values twice the critical DModX values (red line) are outliers.

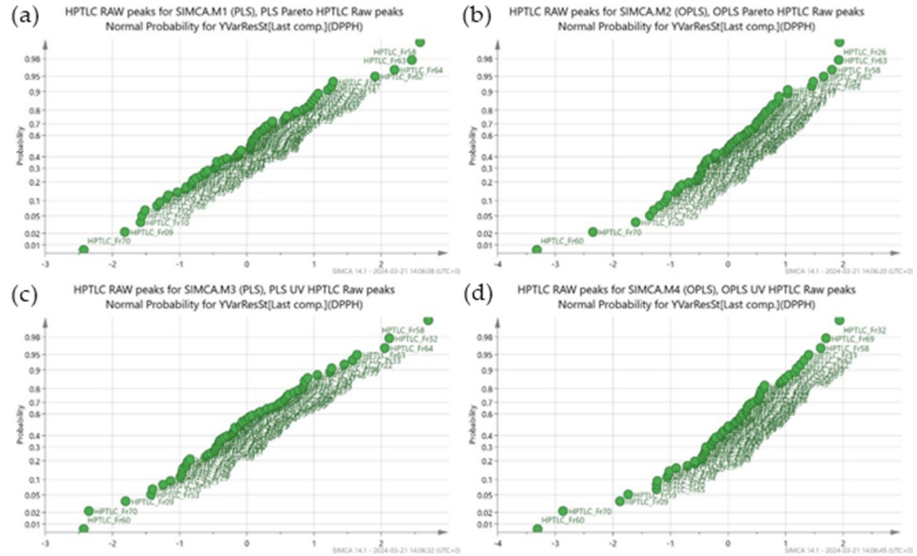

Figure S15. Normal probability plots of residuals of the (a) DPPH PLS Par; (b) DPPH OPLS Par; (c) DPPH PLS UV and (d) DPPH OPLS UV models.

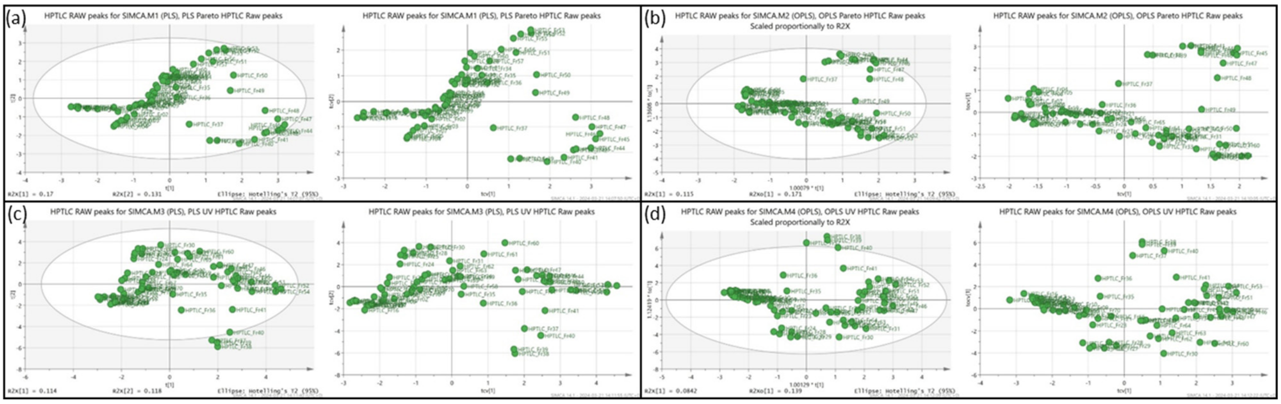

Figure S16. Scores and cross-validation Scores (CV-Scores) plots, respectively, of the (a) DPPH PLS Par; (b) DPPH OPLS Par; (c) DPPH PLS UV and (d) DPPH OPLS UV models.

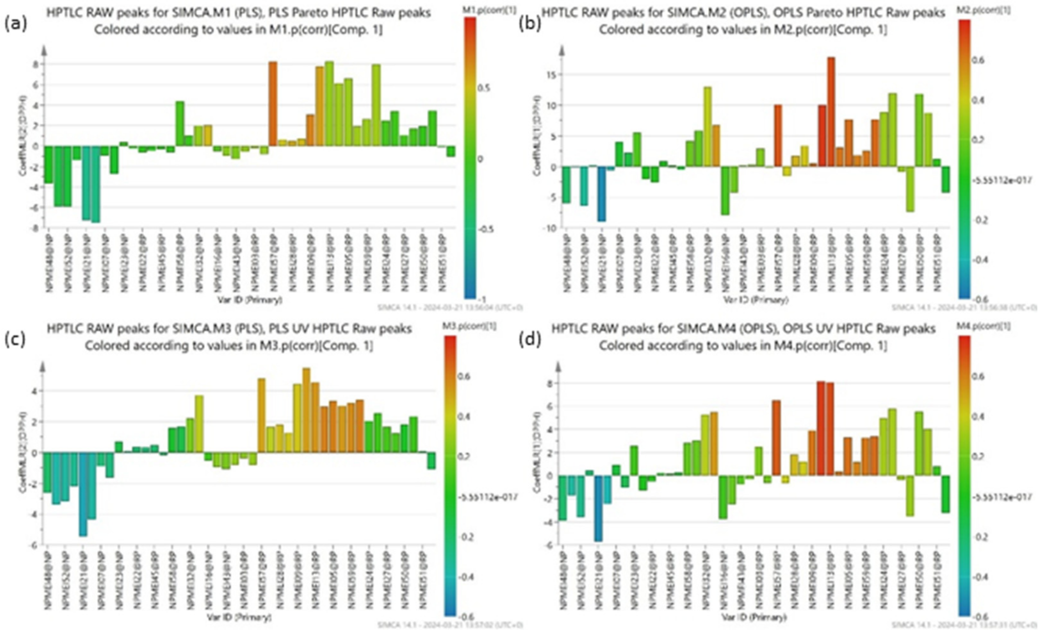

Figure S17. Coefficient plots of the models (a) DPPH PLS Par; (b) DPPH OPLS Par; (c) DPPH PLS UV and (d) DPPH OPLS UV.

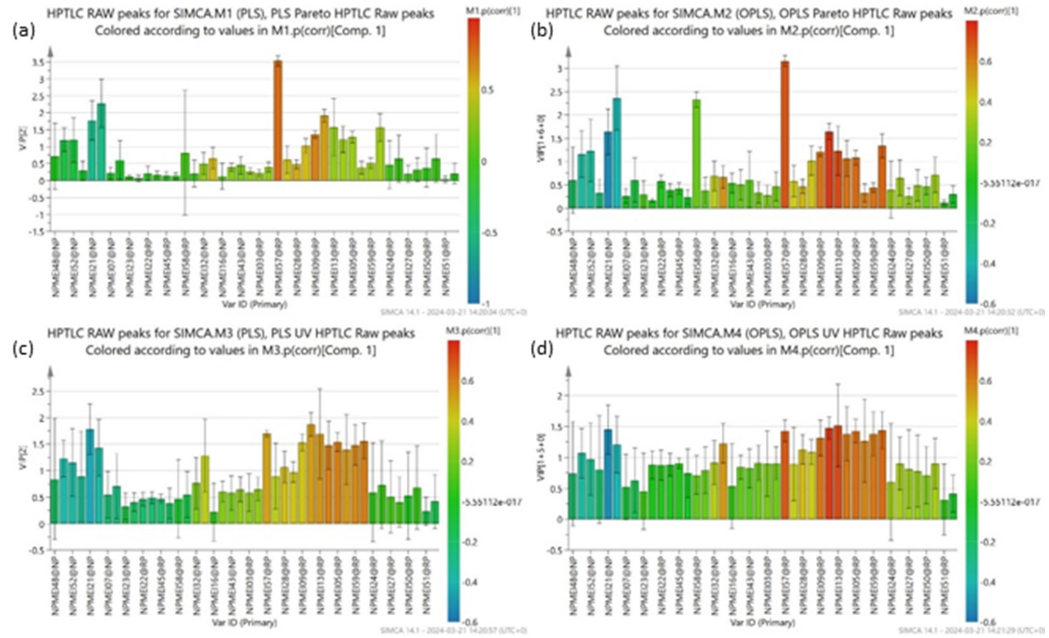

Figure S18. VIP plots of the models (a) DPPH PLS Par; (b) DPPH OPLS Par; (c) DPPH PLS UV and (d) DPPH OPLS UV.

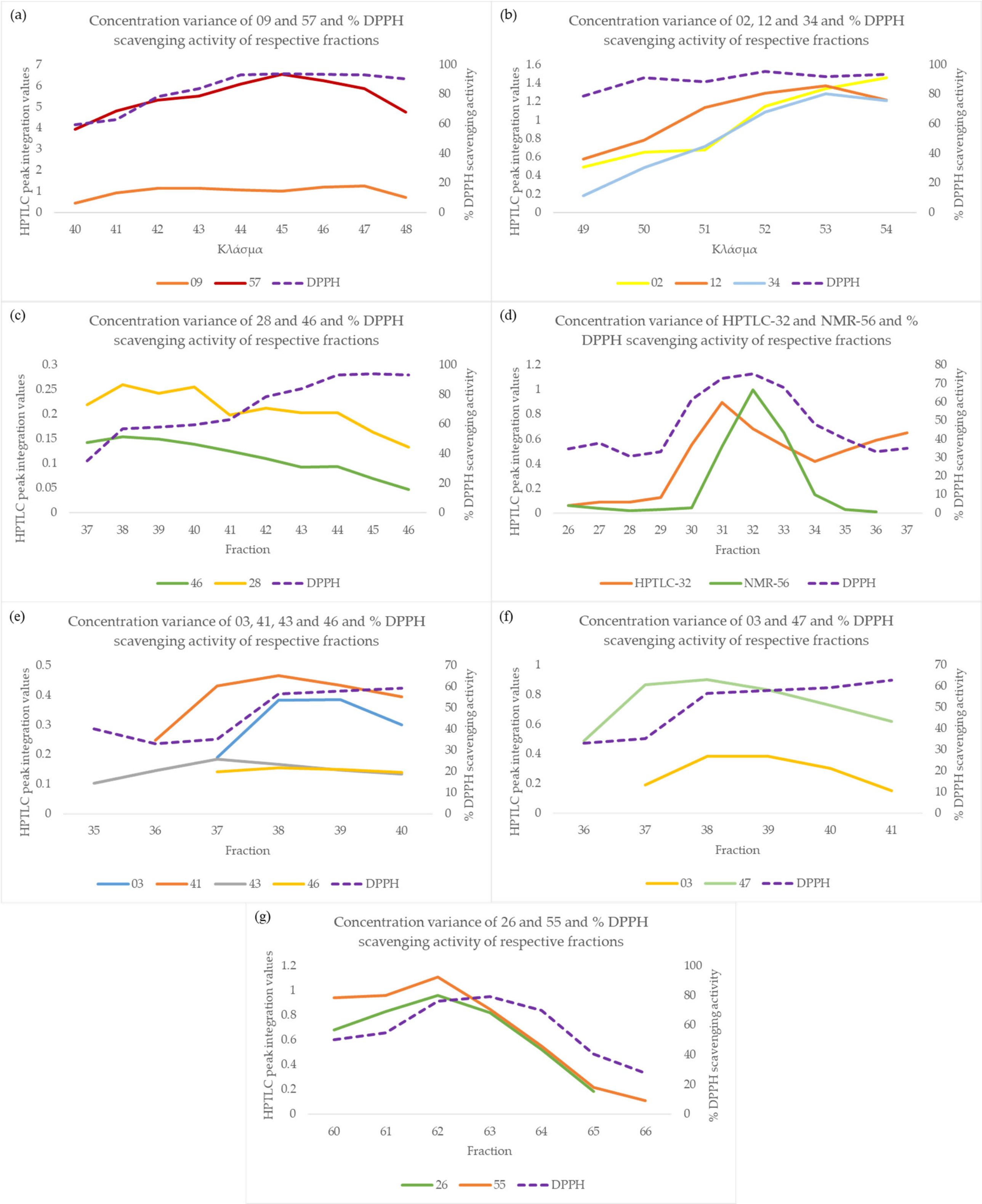

Figure S19. Concentration variance of compounds (a) 09 and 57 (Fr40-48); (b) 02, 12 and 34 (Fr49-54); (c) 28 and 46 (Fr37-46); (d) HPTLC-32 and NMR-56 (Fr26-37); (e) 03, 41, 43 and 46 (Fr35-40); (f) 03 and 47 (Fr36-41) and (g) 26 and 55 (Fr60-66) and % DPPH scavenging activity of respective fractions.

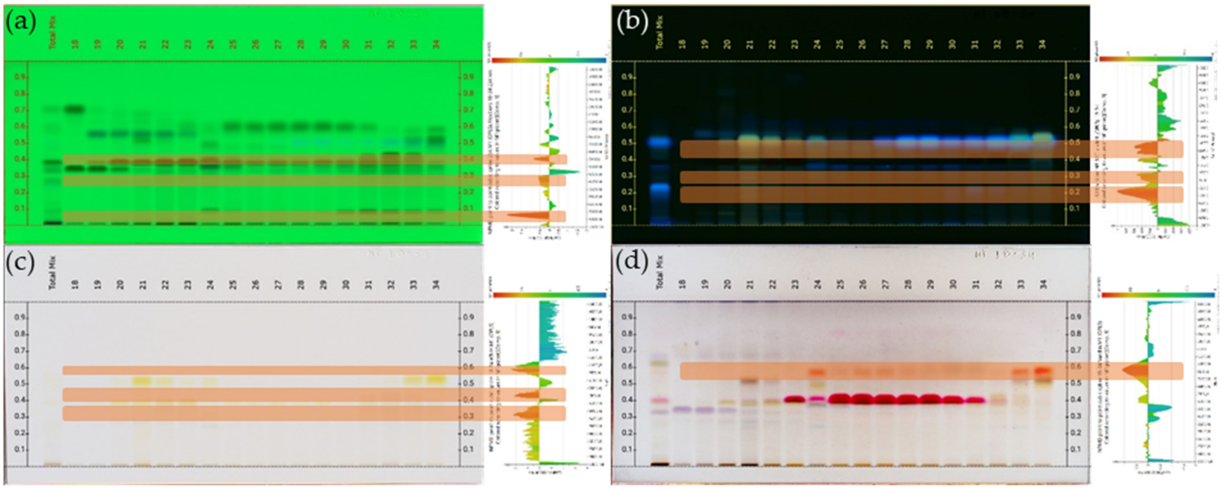

Figure S20. HPTLC chromatograms juxtaposed with the corresponding coefficient plots of DPPH scavenging activity of fractions Fr18-34 in normal phase, obtained from the multivariate analysis (a) at 254 nm; (b) at 366 nm; (c) at visible light and (d) in visible light after derivatization with sulfuric vanillin reagent. Examples of spots that appear to be highly correlated with the activity are listed in a red box.

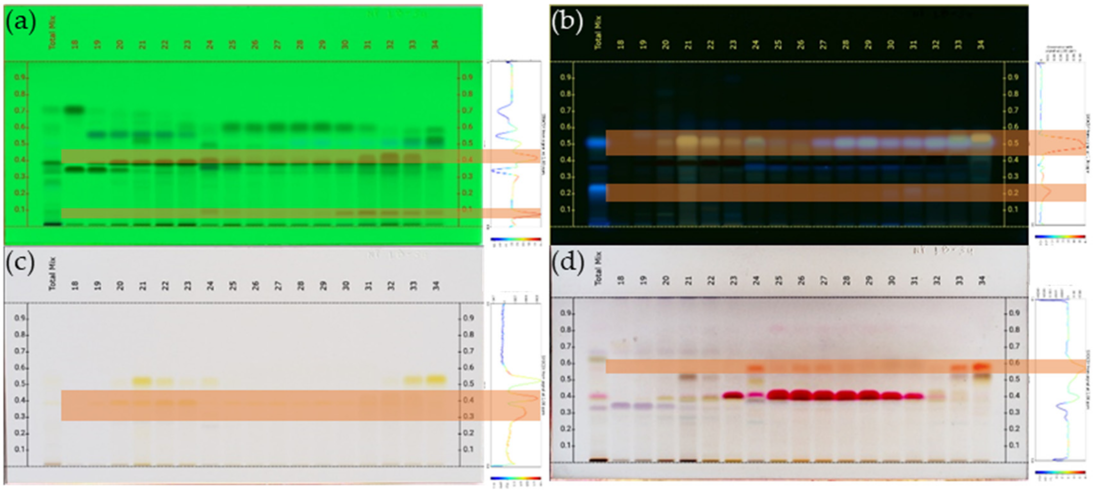

Figure S21. HPTLC chromatograms juxtaposed with the corresponding HetCA plots of DPPH scavenging activity of fractions Fr18-34 in normal phase, obtained from the heterocovariance approach (a) at 254 nm; (b) at 366 nm; (c) at visible light and (d) in visible light after derivatization with sulfuric vanillin reagent. Examples of spots that appear to be highly correlated with the activity are listed in a red box.
